# Density functional theory investigation of oxygen-deficient SnO_2-x_: Optoelectronic property tuning and hydrogen gas interaction

**DOI:** 10.1371/journal.pone.0357265

**Published:** 2026-09-02

**Authors:** Manoj Kumar, Purnendu Shekhar Pandey, M. Sudhakara Reddy, Anita Gehlot, Jaishanker Prasad Keshari, Akash Kumar Pradhan, Gyanendra Kumar Singh, Balkeshwar Singh

**Affiliations:** 1 Department of Electronics and Communication Engineering, MLR Institute of Technology, Hyderabad, India; 2 Department of Electronics and Communication Engineering, G.L. Bajaj Institute of Technology and Management, Greater Noida, UP, India; 3 Department of Physics & Electronics, JAIN (Deemed to be University), Bangalore, Karnataka, India; 4 Uttaranchal Institute of Technology, Uttaranchal University, Dehradun, India; 5 Government Engineering College, Sheikpura, Bihar Engineering University, Patna, India; 6 Vellore Institute of Technology Chennai, Chennai, Tamil Nadu, India; 7 Faculty of Industrial & Manufacturing Technology & Engineering, Universiti Teknikal Malaysia Melaka, Melaka, Malaysia; 8 Department of Manufacturing Technology, FDRE Technical and Vocational Training Institute, Addis Ababa, Ethiopia; Institute of Biophysics Chinese Academy of Sciences, CHINA

## Abstract

Oxygen-vacancy engineering is an effective strategy for tailoring the electronic, optical, and gas-sensing properties of tin dioxide (SnO_2_). In this work, first-principles Density Functional Theory (DFT) calculations were performed to systematically investigate the influence of oxygen vacancies on the optoelectronic characteristics and hydrogen adsorption behavior of pristine SnO_2_ and oxygen-deficient SnO_2-x_. Oxygen-deficient structures were considered with SnO_2-x_ (x = 0.02, 0.06, and 0.10), corresponding to oxygen-vacancy concentrations of 1%, 3%, and 5%, respectively. A 5 × 5 × 1 rutile SnO_2_ supercell containing 50 Sn and 100 O atoms was employed, and oxygen-deficient models were generated by removing 1, 3, and 5 oxygen atoms, yielding Sn₅₀O₉₉, Sn₅₀O₉₇, and Sn₅₀O₉₅ configurations. Electronic structure calculations reveal that oxygen vacancies introduce donor-like defect states near the conduction band, increasing carrier concentration and modifying the density of states. Projected density of states analysis confirms that these defect states originate primarily from the hybridization of Sn-derived orbitals with perturbed O 2p states surrounding the vacancy sites. The calculated optical properties demonstrate that oxygen deficiency enhances the refractive index, extinction coefficient, dielectric response, optical absorption, and reflectance, with the 3% oxygen-vacancy model exhibiting the most favorable overall optical performance. Hydrogen adsorption calculations performed on the thermodynamically stable SnO_2_(110) surface indicate that oxygen vacancies strengthen H_2_ adsorption by creating electronically active surface sites and facilitating charge transfer.

## 1. Introduction

Tin dioxide (SnO_2_) is a wide band gap semiconductor that has been extensively investigated for applications in optoelectronic devices, transparent conducting oxides (TCOs), photocatalysis, and gas sensing technologies [1,2]. Its wide band gap, high electron mobility, excellent thermal stability, and strong resistance to chemical corrosion make SnO_2_ a suitable material for operation in harsh environments [3]. In optoelectronic applications, SnO_2_ is valued for its high optical transparency in the visible region combined with good electrical conductivity [4], while in gas sensing devices it serves as an active sensing layer capable of interacting with various reducing and oxidizing gases. Despite these advantages, pristine SnO_2_ often suffers from limited gas sensitivity, slow response–recovery times, and poor selectivity, particularly at lower operating temperatures [5]. These limitations primarily arise from its relatively low density of surface-active sites and insufficient charge transfer during gas adsorption, which restrict its overall sensing performance [6].

Defect engineering has emerged as an effective strategy for overcoming these limitations by tailoring the structural, electronic, and optical properties of semiconductor metal oxides. Oxygen vacancies are among the most important intrinsic point defects in metal oxide semiconductors and play a decisive role in controlling their structural, electronic, and optical characteristics [2]. In SnO_2-x_, oxygen vacancies act as defects that introduce localized electronic states near the conduction band, thereby increasing free carrier concentration and electrical conductivity. The deliberate introduction of oxygen deficiency can significantly modify the band structure, reduce the effective band gap, and enhance light–matter interaction [7]. Furthermore, oxygen vacancies create unsaturated coordination sites on the SnO_2-x_ surface, which serve as energetically favorable adsorption centres for gas molecules. In particular, oxygen-deficient SnO_2-x_ has demonstrated strong potential for hydrogen (H_2_) gas sensing, as the presence of vacancies facilitates enhanced adsorption, dissociation, and charge transfer between H_2_ molecules and the SnO_2_ surface [8]. These defect-induced interactions lead to measurable changes in the electronic and optical response of the material, thereby improving sensor sensitivity [5].

DFT offers a powerful and reliable theoretical framework for investigating defect-driven modifications in materials at the atomic and electronic levels. Through first-principles calculations, DFT enables detailed analysis of electronic structure, density of states, and optical response as a function of defect concentration [9]. In the present study, DFT calculations are systematically employed to examine the influence of controlled oxygen vacancy concentrations on the optoelectronic properties of SnO_2_ [10]. Key optical parameters, including refractive index, extinction coefficient, dielectric function, reflectance, and absorption coefficient, are analyzed and compared with pristine SnO_2_. In addition, the Adsorption Locator tool is utilized to explore the interaction mechanisms and sensing behavior of H_2_ gas molecules on both pristine and oxygen-deficient SnO_2_ surfaces [11]. This combined electronic, optical, and adsorption analysis provides comprehensive theoretical insight into the role of oxygen vacancies in optimizing SnO_2_ for advanced optoelectronic and hydrogen gas sensing applications.

## 2. Computational methodology

### 2.1. Structural modeling

**Fig 1:**
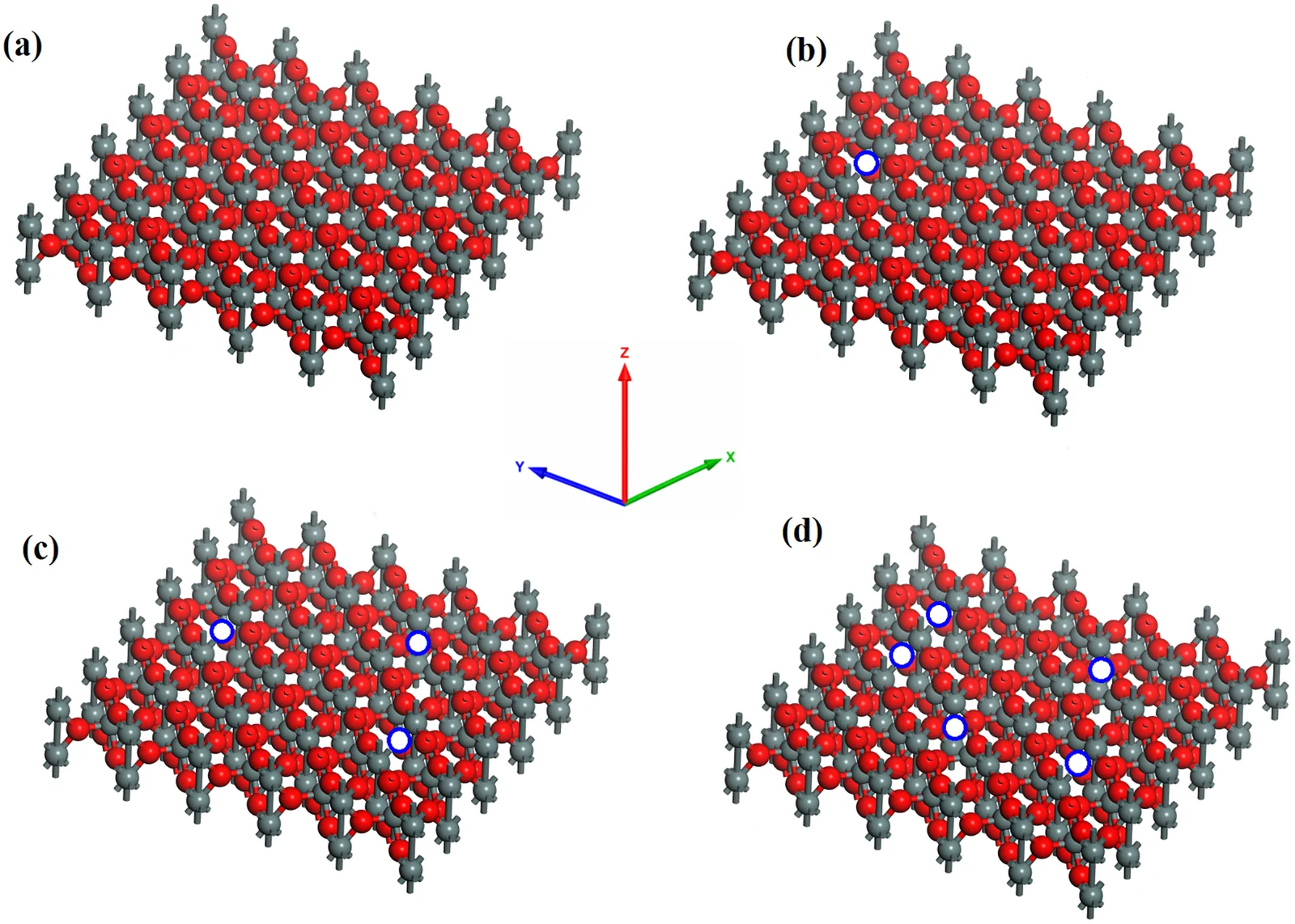
Atomic structural models of (a) pristine SnO_2_, (b) SnO_2-_x (1% oxygen-deficient), (c) SnO_2-x_ (3% oxygen-deficient), and (d) SnO_2-x_ (5% oxygen-deficient).

The atomic structures of pristine SnO_2_ and oxygen-deficient SnO_2-x_ are shown in Fig 1(a–d). The atomic structure of pristine SnO_2_ has been shown in Fig 1(a) which is based on its tetragonal rutile phase. To model oxygen-deficient SnO_2-x_, a supercell containing approximately 100 oxygen atoms (represented by red spheres) and tin (Sn) atoms (represented by grey spheres) was constructed. Oxygen-deficient structures of SnO_2-x_ (x = 0.02, 0.06, and 0.10) are generated by systematically removing 1, 3, and 5 oxygen atoms from the optimized supercell, yielding compositions of Sn₅₀O₉₉, Sn₅₀O₉₇, and Sn₅₀O₉₅, which corresponds to 1%, 3%, and 5% oxygen-vacancy concentrations. When normalized per Sn atom, these correspond to the compositions, SnO_1.98_, SnO_1.94_, and SnO_1.90_, respectively. The oxygen vacancies are represented by blue rings in Fig 1(b–d). All structures were geometrically optimized to obtain stable configurations prior to electronic and optical property calculations. It may be mentioned here that the oxygen vacancy positions are chosen as representative oxygen-deficient configurations while avoiding unrealistically strong vacancy–vacancy interactions caused by placing vacancies immediately adjacent to one another within the periodic supercell [12]. This choice was motivated by the fact that oxygen vacancies in SnO_2_ can exhibit site dependence, defect–defect interaction, and concentration-dependent stability, and different vacancy geometries may lead to different local lattice relaxations and electronic states [13]. In the present calculations, the vacancy sites for the multi-vacancy models were chosen to be relatively dispersed rather than strongly clustered, so as to reduce unrealistically strong local vacancy–vacancy coupling within the finite periodic cell and to better represent a dilute-to-moderate oxygen-deficient regime [14]. Furthermore, the oxygen-deficient SnO_2_ systems are modeled using neutral supercells, and therefore the oxygen vacancies were treated as neutral oxygen vacancies within the periodic DFT framework rather than as explicitly charged defects such as V_O_^+^ or V_O_^2+^. This choice is consistent with the scope of the present work, which is focused on comparing the structural, electronic, and optical behavior of representative oxygen-deficient SnO_2_ models at different vacancy concentrations, rather than performing a full charged-defect thermodynamic analysis.

### 2.2. DFT setup

To systematically investigate the structural, electronic, and optical properties of pristine and oxygen-deficient SnO_2_, the crystal structure of SnO_2_ was initially constructed based on its tetragonal rutile phase. DFT calculations were performed using the CASTEP module of Materials Studio (BIOVIA) to evaluate the influence of oxygen vacancies on the optoelectronic characteristics of SnO_2-x_ systems. A supercell containing approximately 100 oxygen atoms was generated to model oxygen-deficient SnO_2_, and controlled oxygen vacancies were introduced by selectively removing oxygen atoms to obtain SnO_2-x_ (x = 0.02, 0.06, and 0.10), corresponding to oxygen-vacancy concentrations of 1%, 3%, and 5%, respectively. These defect concentrations are chosen to systematically analyze the effect of vacancy-induced defects on the material properties. To investigate the role of oxygen vacancies in SnO_2_, two complementary computational models are employed. Bulk supercell calculations have been performed to evaluate the effect of oxygen vacancies on the intrinsic structural, electronic, and optical properties of SnO_2_. These calculations provide information on vacancy-induced modifications in the electronic structure, such as defect states and changes in the density of states. In addition, surface slab models were constructed to investigate H_2_ adsorption behavior and the interaction between gas molecules and oxygen-deficient SnO_2_ surfaces, which is directly related to gas-sensing performance. The combination of bulk and surface calculations allows the relationship between vacancy-induced electronic structure modification and surface adsorption activity to be systematically analyzed. It may be mentioned here that the electronic structure calculations have been carried out within the framework of the generalized gradient approximation (GGA) using the Perdew–Burke–Ernzerhof (PBE) exchange–correlation functional, along with norm-conserving pseudopotentials for all constituent elements. Grimme's D3(BJ) dispersion correction correction was incorporated to account for long-range van der Waals interactions [15]. Brillouin-zone sampling for the bulk supercell calculations was performed using a 2 × 2 × 1 Monkhorst–Pack k-point mesh, while a plane-wave cutoff energy of 830 eV was employed throughout the calculations. For the thin-film model, the 2 × 2 × 1 k-point mesh is appropriate because the supercell is expanded in the in-plane directions, while the c-axis is the out-of-plane direction containing the vacuum region. Thus, no additional k-point sampling along the c-axis was used. To verify the adequacy of the selected k-point mesh, k-point convergence tests were performed by comparing the calculated total energy and relevant electronic properties using progressively denser meshes. The results showed that the 2 × 2 × 1 mesh provides converged results within the required accuracy, with no significant change in the calculated quantities upon further increasing the k-point density. Moreover, this k-point mesh density (2 × 2 × 1) is commonly employed for geometry optimization and electronic structure calculations of large oxide thin film supercells, where Brillouin-zone folding substantially reduces the k-point sampling required relative to primitive cells. We acknowledge here that a denser k-point mesh could further improve the accuracy of convergence.

Geometry optimization has been carried out until the convergence criteria of 0.03 eV Å^−1^ for the maximum force, 0.05 GPa for the maximum stress, 0.001 Å for the maximum atomic displacement, and 1 × 10^−6^ eV atom^−1^ for the self-consistent field (SCF) energy were satisfied. The maximum force, maximum stress, and maximum atomic displacement were set to 0.03 eV/Å, 0.05 GPa, and 0.001 Å, respectively. The self-consistent field (SCF) convergence tolerance was fixed at 10^−6^ eV/atom to ensure high precision in electronic structure calculations [16]. The adsorption configurations of hydrogen (H_2_) molecules on pristine and oxygen-deficient SnO_2_ surfaces have been generated using the Adsorption Locator module of Materials Studio, followed by structural optimization and adsorption energy calculations using DFT methods.

### 2.3. Formation energy and structural stability

To evaluate the energetic feasibility of oxygen-vacancy formation and confirm the stability of the constructed SnO_2-x_ models, the oxygen-vacancy formation energies have been calculated for the different defect concentrations. The formation energies which are found out to be 3.1 eV, 2.7 eV, and 2.3 eV for the 1%, 3%, and 5% oxygen vacancy models, respectively. These values are in good agreement with previously reported DFT studies, which typically report oxygen-vacancy formation energies [17]. The positive formation energies indicate that all oxygen-deficient configurations are energetically feasible. The slight decrease in formation energy with increasing vacancy concentration suggests that the formation of additional vacancies becomes progressively more favorable because of defect-defect interactions and local structural relaxation [18]. Furthermore, all structures were fully optimized until the convergence criteria are satisfied. The optimized structures retained the rutile SnO_2_ framework without any structural collapse, although local distortions around the vacancy sites became more pronounced at higher vacancy concentrations. These results demonstrate that the investigated oxygen-deficient SnO_2_ models are both energetically and structurally stable, supporting the reliability of the calculated electronic and optical properties.

### 2.4. Optoelectronic property calculations

The optoelectronic properties of pristine and oxygen-deficient SnO_2_ systems have been systematically investigated to obtain a comprehensive understanding of the influence of oxygen vacancies on their electronic and optical characteristics [19]. The properties computed included the refractive index, extinction coefficient, real and imaginary parts of the dielectric function, reflectance, and absorption coefficient. The refractive index and extinction coefficient are calculated to quantify the propagation characteristics of light within the material, specifically indicating the extent of light attenuation as it traverses the SnO_2_ lattice. The real component of the dielectric function represents the material’s capacity to store electromagnetic energy, while the imaginary component reflects the energy loss resulting from absorption processes [20]. Comparative analysis of these wavelength-dependent optical parameters have been performed to establish the relationship between oxygen-vacancy concentration, electronic structure modification, and optical behaviour for its potential applications in optoelectronic and photonic devices.

### 2.5. Density of states analysis

The electronic structure of pristine SnO_2_ and oxygen-deficient SnO_2-x_ have been investigated through total density of states (DOS) and projected density of states (PDOS). The DOS calculations are performed using the optimized spin-polarized bulk structures to evaluate the influence of oxygen-vacancy defects on the distribution of electronic states near the Fermi level. In addition, PDOS analysis have been carried out to identify the orbital contributions of Sn and O atoms to the valence band, conduction band, and vacancy-induced defect states [21].

Comparative analysis between pristine and oxygen-deficient SnO_2_ was performed to examine the evolution of donor-like defect states introduced by oxygen vacancies and their influence on the electronic structure. The DOS and PDOS results provide insight into the origin of the vacancy-induced electronic states, the modification of band-edge characteristics, and the hybridization between Sn and O orbitals. These electronic structure changes are directly correlated with the calculated optical properties, including the dielectric response, refractive index, extinction coefficient, and optical absorption. Consequently, the combined DOS and PDOS analyses provide a comprehensive understanding of how controlled oxygen-vacancy concentrations can be employed to tailor the electronic and optoelectronic performance of SnO_2_ for advanced optoelectronic and hydrogen gas-sensing applications.

### 2.6. Adsorption locator study for H_2_ gas sensing

The interaction between hydrogen (H_2_) molecules and pristine as well as oxygen-deficient SnO_2_ surfaces have been investigated using the Adsorption Locator module implemented in Materials Studio. Adsorption calculations were performed on the thermodynamically stable SnO_2_(110) surface [22], which was constructed from the optimized bulk structure. The surface slab consisted of five atomic layers separated by a 15 Å vacuum region to eliminate interactions between periodic images. Oxygen-deficient surface models are generated by introducing neutral oxygen vacancies into the surface layer, corresponding to the defect concentrations considered in the bulk calculations.

## 3. Results and discussion

### 3.1. Electronic structure analysis using DOS and PDOS

#### 3.1.1. Density of states (DOS).

**Fig 2:**
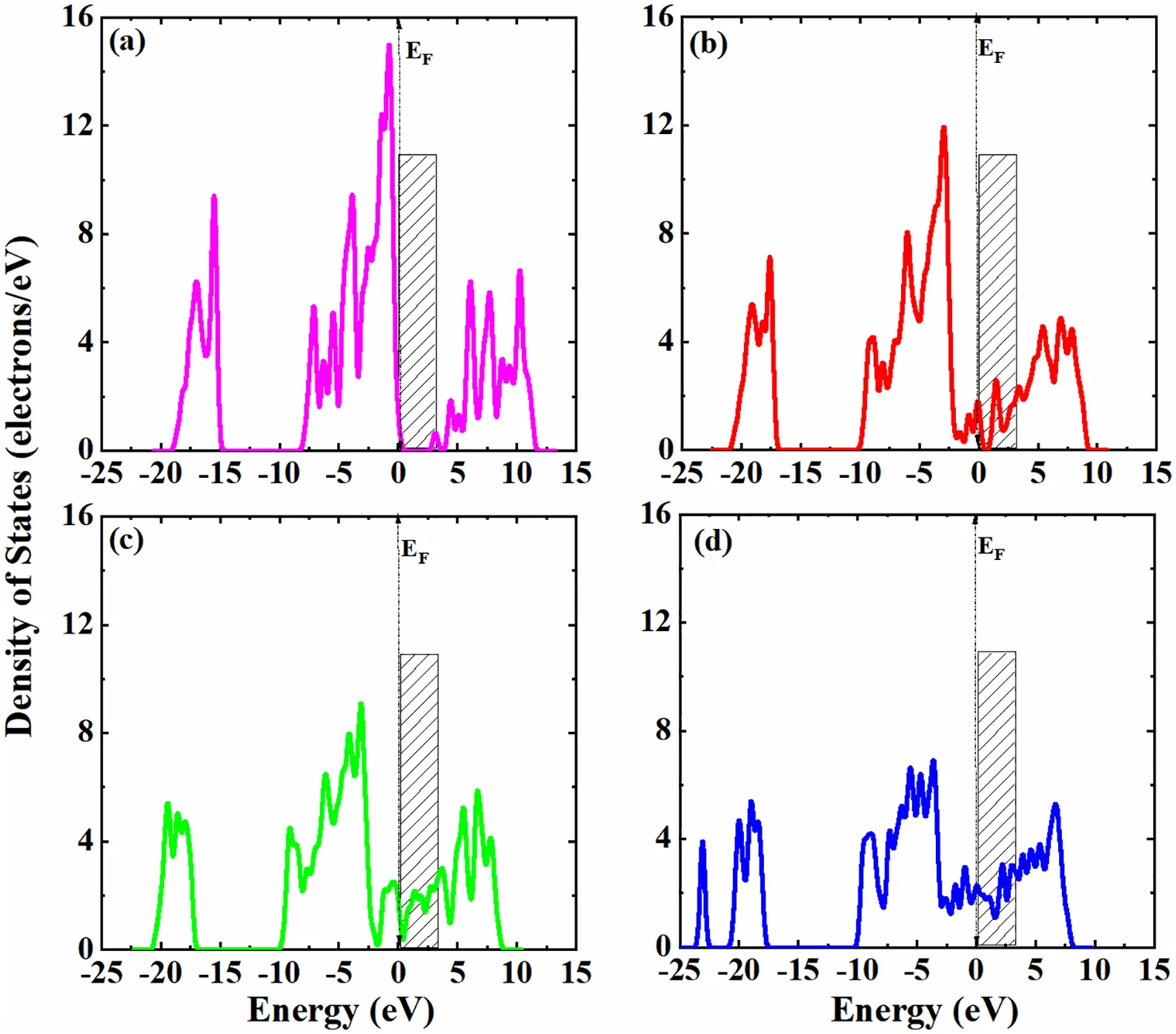
The total density of states for (a) pristine SnO_2_, (b) SnO_2-x_ (1% oxygen-deficient), (c) SnO_2-x_ (3% oxygen-deficient), and (d) SnO_2-x_ (5% oxygen-deficient).

The Fig 2 represents the total density of states of (a) pristine SnO_2_, (b) SnO_2-x_ with 1% oxygen vacancy, (c) SnO_2-x_ with 3% oxygen vacancy, and (d) SnO_2-x_ with 5% oxygen vacancy. The shaded region represents the forbidden energy gap, while the Fermi level is set at 0 eV which is denoted by E_F._ It is observed from Fig 2(a), pristine SnO_2_ exhibits a well-defined forbidden energy gap with no electronic states inside the gap, confirming its intrinsic semiconducting nature and the absence of defect-induced electronic states. The calculated forbidden energy gap is ~ 2.2 eV, which is lower than the experimentally reported value of ~3.6 eV [23].

The introduction of oxygen vacancies significantly modifies the electronic structure of SnO_2_. For the 1% oxygen-deficient SnO_2_ (Fig 2(b)), defect-induced electronic states emerge within the forbidden energy gap, particularly close to the conduction band edge. These localized states originate from oxygen vacancy defects and act as shallow donor levels, reducing the effective band gap and facilitating electron excitation into the conduction band. Consequently, the electron concentration increases, leading to enhanced electrical conductivity compared with pristine SnO_2_ [24].

With a further increase in oxygen vacancy concentration to 3% (Fig 2(c)), the density of defect states within the forbidden energy gap increases noticeably. The defect levels become more uniformly distributed across the gap, indicating stronger perturbation of the electronic structure. The higher density of donor states provides additional pathways for electron transport and promotes efficient charge transfer [25]. The increased overlap between the defect states and the conduction band also indicates enhanced electronic conductivity resulting from the higher oxygen vacancy concentration.

For the 5% oxygen-deficient SnO_2_ (Fig 2(d)), the defect states become even more pronounced and occupy a larger portion of the forbidden energy gap. The increased density of localized electronic states suggests a substantial increase in donor concentration, which can significantly improve electrical conductivity by supplying more free electrons [26]. However, excessive oxygen vacancies may also introduce highly localized defect centers that increase carrier scattering and electron–hole recombination, thereby reducing carrier mobility despite the higher electron concentration. The excessive defect density may also increase carrier recombination, potentially limiting device performance [27].

#### 3.1.2. Projected density of states (PDOS).

**Fig 3:**
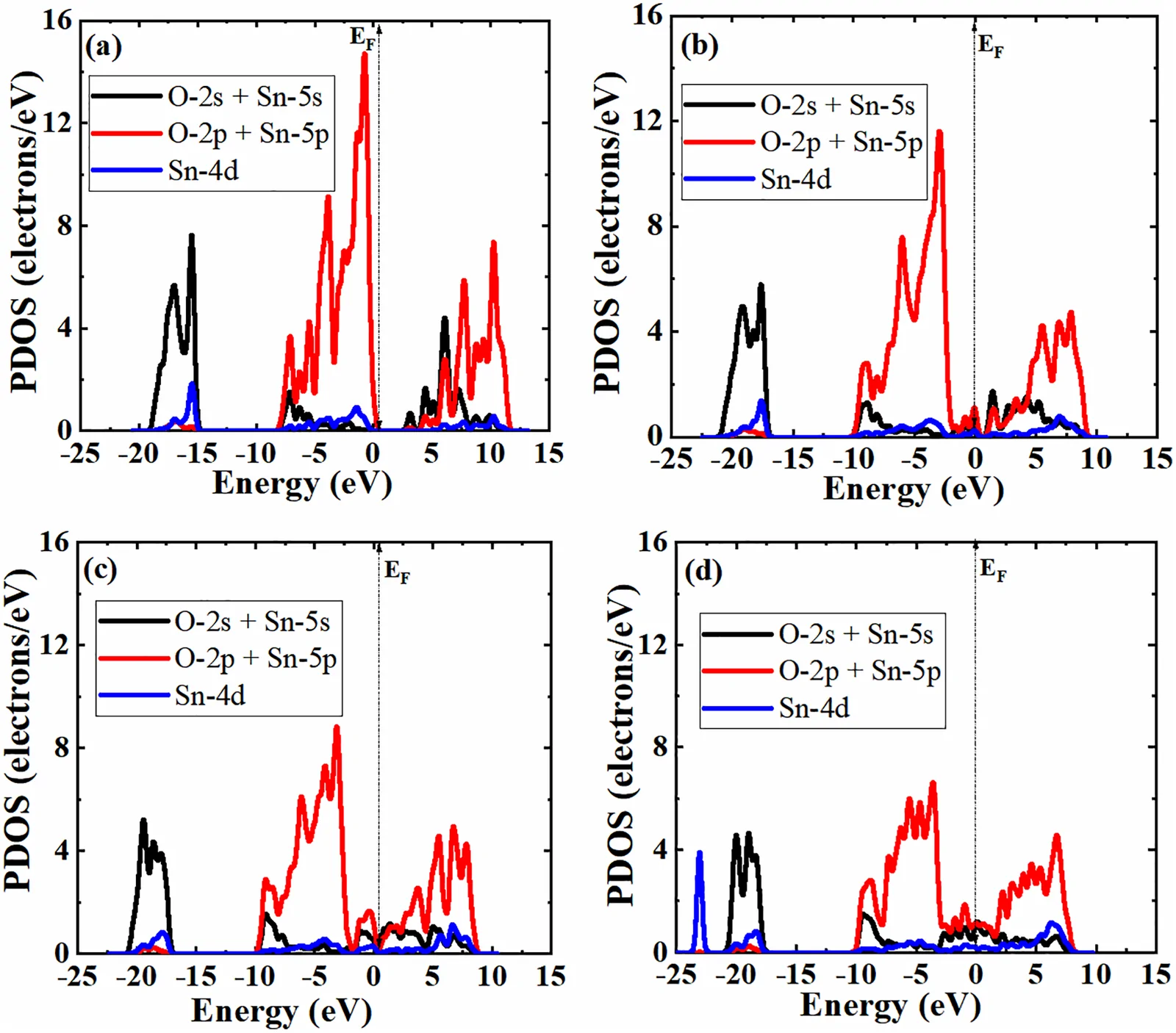
The projected density of states for (a) pristine SnO_2_, (b) SnO_2-x_ (1% oxygen-deficient), (c) SnO_2-x_ (3% oxygen-deficient), and (d) SnO_2-x_ (5% oxygen-deficient).

To further elucidate the origin of the electronic states observed in the total density of states (DOS), the projected density of states (PDOS) of pristine and oxygen-deficient SnO_2_ have been analyzed, as shown in Fig 3(a–d). The Fig 3(a) represents pristine SnO_2_, while Fig 3(b), 3(c), and 3(d) correspond to SnO_2-x_ with 1%, 3%, and 5% oxygen vacancies, respectively. The PDOS is resolved into the combined O-2s + Sn-5s, O-2p + Sn-5p, and Sn-4d contributions, with the Fermi level (E_F_) set to 0 eV.

For pristine SnO_2_ (Fig 3(a)), the deep valence-band region (approximately −20 to −15 eV) is mainly associated with the O-2s + Sn-5s states, whereas the upper valence-band region (approximately −8 eV to E_F_) is dominated by the O-2p + Sn-5p states. The Sn-4d contribution is mainly confined to the deeper valence region. The pronounced overlap between the O- and Sn-derived states indicates strong orbital interaction and contributes to the electronic structure of pristine SnO_2_.

With increasing oxygen-vacancy concentration from 1% to 3% and 5% [Figs 3(b–d)], a progressive redistribution of the combined orbital states is observed, particularly near E_F_. The increasing oxygen deficiency perturbs the local Sn–O coordination and modifies the orbital hybridization, resulting in additional electronic states near the Fermi level. These changes are attributed to oxygen-vacancy-induced defect states associated with the altered electronic structure of the neighboring Sn atoms [28].

At 5% oxygen vacancy (Fig 3(d)), the modification of the PDOS near E_F_ is more pronounced, reflecting the stronger perturbation of the Sn–O bonding environment and increased local structural distortion around vacancy sites. Overall, the PDOS of pristine and oxygen deficient SnO_2_ demonstrates that oxygen deficiency progressively modifies the electronic structure of SnO_2_, particularly near the Fermi level, through redistribution of the O-2s + Sn-5s, O-2p + Sn-5p, and Sn-4d states. The resulting vacancy-induced electronic states can enhance carrier concentration and facilitate charge transfer, which is favorable for gas-sensing applications. However, excessive vacancy concentration may also increase structural disorder and carrier scattering, suggesting that an intermediate oxygen-vacancy concentration could provide a favorable balance between carrier generation and mobility.

### 3.2. Optical properties

#### 3.2.1. Refractive index (n) vs wavelength.

**Fig 4:**
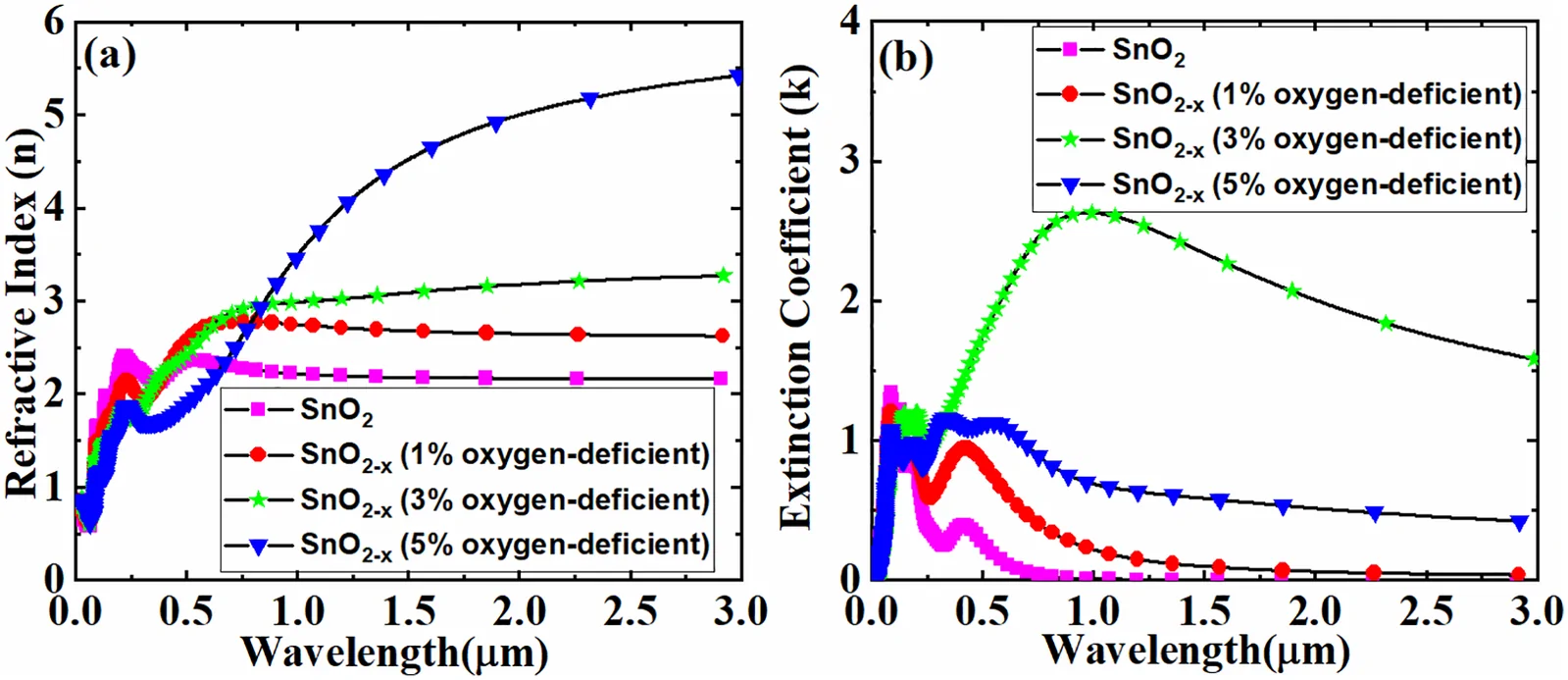
Refractive index and extinction coefficient of pristine and oxygen-deficient SnO_2-x_.

The variation of the refractive index (n) as a function of wavelength for pristine SnO_2_ and oxygen-deficient SnO_2_ ₋ ₓ (1%, 3%, and 5%) is shown in Fig 4(a). Pristine SnO_2_ exhibits a relatively low and nearly constant refractive index over the investigated wavelength range, indicating limited optical dispersion. Upon introducing oxygen vacancies, a pronounced enhancement in the refractive index is observed, particularly at higher vacancy concentrations [29]. Pristine SnO_2_ exhibits a nearly constant refractive index of approximately 2.2 in the near-infrared region, consistent with its dielectric semiconductor behaviour. Introducing oxygen vacancies progressively increases the refractive index due to defect-induced modifications of the electronic structure and enhanced electronic polarizability. The 1% and 3% oxygen-vacancy models exhibit moderate increases in refractive index, reaching approximately 2.7 and 3.3, respectively. In contrast, the 5% oxygen-vacancy model shows a pronounced increase, with the refractive index exceeding 5 at longer wavelengths. This behaviour originates from the substantial increase in donor-like defect states and free-carrier concentration produced by the high density of oxygen vacancies, which significantly enhances the low-energy dielectric response. Such a large refractive index should therefore be interpreted as evidence of the transition toward metallic-like optical behaviour associated with heavy oxygen deficiency [30], rather than as an indication of improved dielectric performance. The high vacancy concentration introduces partially delocalized electronic states and increased carrier density, resulting in strong dispersion and a pronounced increase in the calculated refractive index [31].

#### 3.2.2. Extinction coefficient (k) vs wavelength.

The Fig 4(b) illustrates the wavelength-dependent extinction coefficient (k) of pristine and oxygen-deficient SnO_2-x_. The extinction coefficient represents the optical energy loss within the material due to absorption and is directly related to the imaginary part of the complex dielectric function. Pristine SnO_2_ exhibits a relatively low extinction coefficient over the investigated wavelength range, reflecting its wide-band-gap semiconducting nature and weak absorption in the visible and near-infrared regions. The introduction of oxygen vacancies significantly modifies the optical absorption behaviour by generating donor-like defect states within the band gap [32]. The oxygen-vacancy (1%) model exhibits a moderate increase in the extinction coefficient, indicating enhanced defect-assisted electronic transitions. A further increase in oxygen-vacancy concentration to 3% results in the highest extinction coefficient, reaching a maximum value of approximately 2.6 near 0.8 μm. This enhancement is attributed to the increased density of vacancy-induced electronic states, which promote interband and defect-assisted optical transitions, thereby increasing optical absorption. However, increasing the oxygen-vacancy concentration to 5% leads to a noticeable reduction in the extinction coefficient despite the higher defect density. This behaviour suggests that excessive oxygen vacancies introduce substantial electronic disorder and increased free-carriers, giving rise to metallic-like characteristics and enhanced carrier scattering along with defect scattering, trap-assisted recombination, and reduced carrier mobility become increasingly important. Consequently, the probability of direct optical transitions decreases, resulting in reduced optical attenuation compared with the 3% oxygen-deficient structure. Several studies on defective SnO_2_ and transparent conducting oxides have shown that oxygen deficiency can increase the n-type carrier density and modify the low-energy dielectric response in the infrared regime. In particular, at sufficiently high carrier concentrations, the free-carrier contribution to the dielectric function becomes important, leading to a strong increase in the imaginary part of the dielectric response at low photon energies and a corresponding change in the refractive index and extinction coefficient. The enhanced optical response near 3 μm in the 3% oxygen-deficient SnO_2_ model as follows: increasing oxygen-vacancy concentration introduces donor/defect states and raises the density of defect-associated carriers, which strengthens low-energy intraband or defect-assisted electronic excitations in the infrared region. This additional carrier contribution alters the complex dielectric function, and hence can produce a pronounced increase in the calculated refractive index and extinction coefficient in the mid-infrared. We therefore interpret the feature as a vacancy-induced enhancement of low-energy optical response arising from defect-mediated carrier contributions and free-carrier dielectric screening**,** rather than claiming a true localized plasmon resonance. These results indicate that a moderate oxygen-vacancy concentration provides the most effective balance between defect-induced optical absorption and electronic transport, whereas excessive oxygen deficiency alters the optical response through increased carrier scattering and partial metallic behaviour [33].

#### 3.2.3. Real part of dielectric function (Re(ε)) vs wavelength.

**Fig 5:**
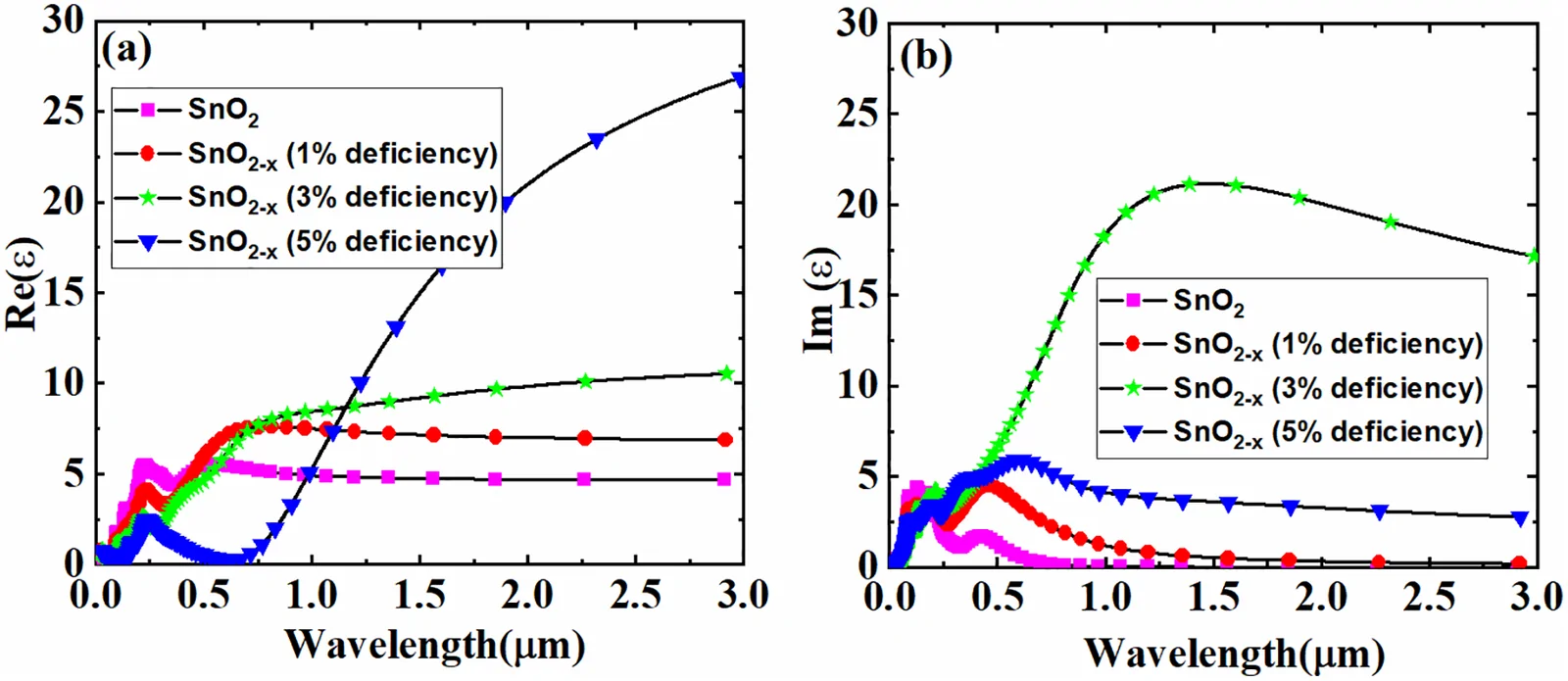
Real and imaginary parts of the dielectric function of pristine SnO_2_ and oxygen-deficient SnO_2__-x_.

Furthermore, the variation of the real part of the dielectric function with wavelength, as shown in Fig 5(a), reveals the significant influence of oxygen-vacancy concentration on the dielectric response of SnO_2_. The real part of the dielectric function represents the material's ability to polarize under an external electromagnetic field and is directly related to its refractive index. Pristine SnO_2_ exhibits a relatively stable dielectric response throughout the investigated wavelength range. The introduction of oxygen vacancies progressively increases Re(ε) due to the formation of donor-like defect states and the associated enhancement in electronic polarizability [34]. The 1% and 3% oxygen-deficient models exhibit moderate increases in Re(ε), whereas the 5% oxygen-deficient model displays a pronounced increase, reaching values above 25 at longer wavelengths. This behaviour is attributed to the substantial increase in free-carrier concentration and defect-state overlap at high oxygen-vacancy concentrations, which strongly enhances the low-energy dielectric response. The exceptionally high Re(ε) observed for the 5% oxygen-deficient structure is therefore indicative of the onset of metallic-like optical behaviour rather than conventional dielectric behaviour, consistent with the electronic structure modifications observed in the DOS analysis [35].

#### 3.2.4. Imaginary part of dielectric function (Im(ε)) vs wavelength.

The corresponding variation in the imaginary part of the dielectric function, Im(ε), is presented in Fig 5(b). Unlike Re(ε), which describes the material's polarization response, Im(ε) represents the optical energy dissipation associated with electronic transitions and is directly related to the absorption of incident electromagnetic radiation. Pristine SnO_2_ exhibits relatively low Im(ε) values over the investigated wavelength range, reflecting the limited number of allowed electronic transitions within its wide band gap. The introduction of oxygen vacancies significantly modifies this behaviour by creating donor-like defect states within the band gap, thereby facilitating defect-assisted optical transitions. Among the investigated models, the 3% oxygen-deficient SnO_2_ exhibits the highest Im(ε), reaching a maximum value of approximately 21 near 1.2 μm. This enhancement is attributed to the increased density of vacancy-induced electronic states, which promotes optical transitions and strengthens light absorption. However, a further increase in the oxygen-vacancy concentration to 5% results in a noticeable reduction in Im(ε), despite the larger number of defects. This reduction indicates that excessive oxygen vacancies introduce significant electronic disorder and increased free-carrier concentration, leading to enhanced carrier scattering and the onset of metallic-like behaviour. As a result, free-carrier effects become increasingly dominant over interband electronic transitions, reducing the optical energy dissipation compared with the 3% oxygen-deficient structure. These observations indicate that a moderate oxygen-vacancy concentration provides the optimum balance between defect-induced electronic transitions and structural stability, thereby enhancing the dielectric and optical response of SnO_2_, whereas excessive oxygen deficiency alters the optical behaviour through increased carrier scattering and partial metallic character.

#### 3.2.5. Absorption coefficient (α) vs wavelength.

**Fig 6:**
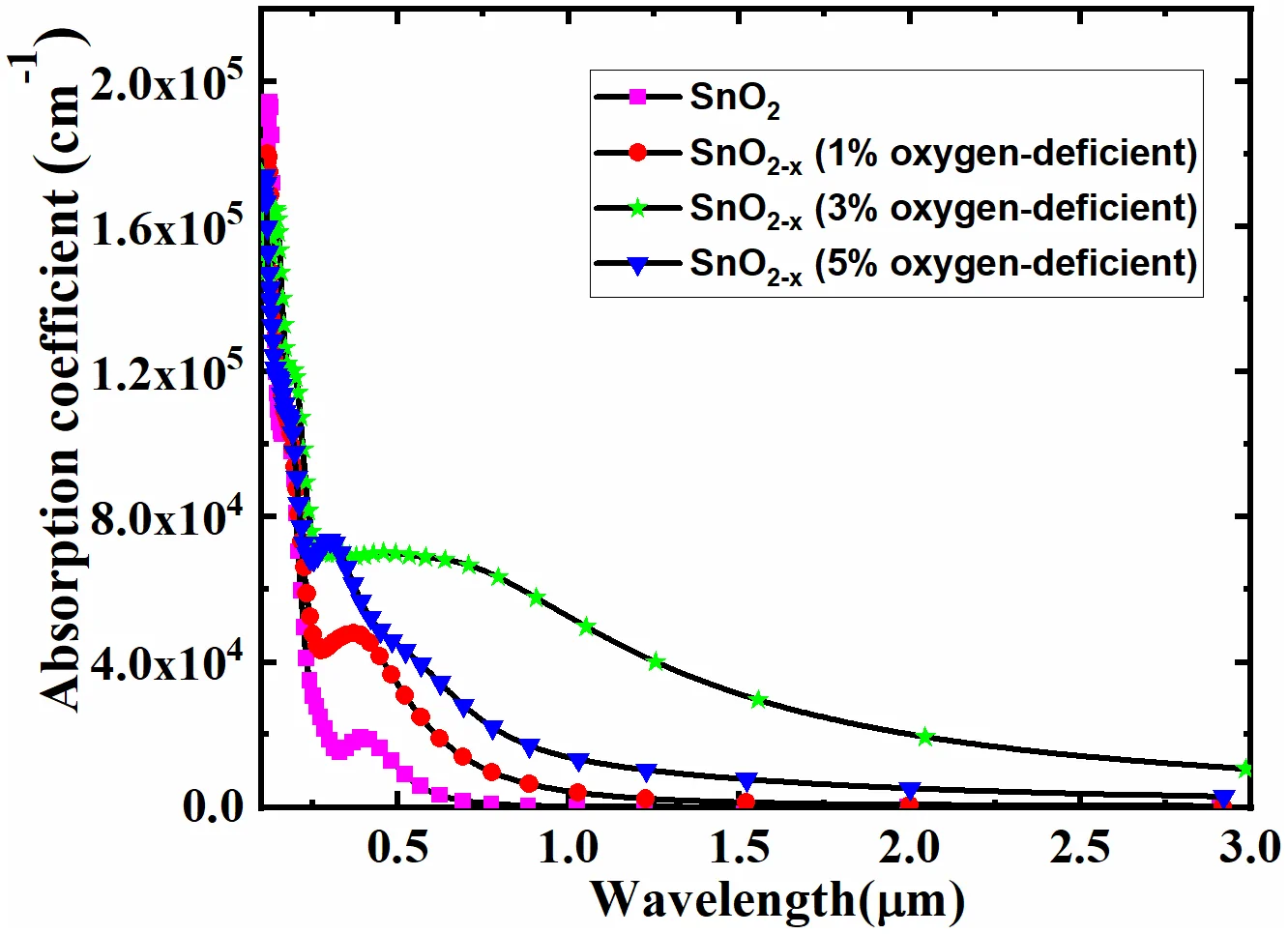
Absorption coefficient spectra of pristine SnO_2_ and oxygen-deficient SnO_2-x_.

The Fig 6 represents the wavelength-dependent absorption coefficient of pristine and oxygen-deficient SnO_2-x_. The absorption coefficient represents the ability of the material to absorb incident photons and is governed by the electronic band structure and the availability of optical transitions [36].

All samples exhibit strong absorption in the ultraviolet region, with absorption coefficients approaching (1.6–1.9) × 10^5^ cm^−1^ at wavelengths below ~0.2 μm, reflecting the intrinsic electronic transitions of SnO_2_. As the wavelength increases, the absorption coefficient decreases for all structures; however, oxygen vacancies significantly modify the absorption behaviour in the visible and near-infrared regions. The pristine SnO_2_ shows a rapid decline in absorption, reaching values below 1 × 10⁴ cm^−1^ beyond 0.6 μm, whereas the 1% oxygen-deficient model maintains a higher absorption coefficient of approximately (4–5) × 10⁴ cm^−1^ near 0.4 μm before gradually decreasing. Among all the investigated structures, the 3% oxygen-deficient SnO_2_ exhibits the strongest optical absorption over a broad wavelength range, with an absorption coefficient of approximately 7 × 10⁴ cm^−1^ between 0.4 and 0.8 μm, which remains above 3 × 10⁴ cm^−1^ even at 1.5 μm. This enhanced absorption is attributed to the increased density of oxygen-vacancy-induced donor states near the conduction band, which facilitate defect-assisted electronic transitions and broaden the optical absorption spectrum. In contrast, the 5% oxygen-deficient SnO_2_ exhibits a lower absorption coefficient than the 3% model, decreasing from approximately 6 × 10⁴ cm^−1^ at 0.4 μm to below 1 × 10⁴ cm^−1^ beyond 1.5 μm. This reduction, despite the higher defect concentration, indicates that excessive oxygen vacancies introduce significant electronic disorder and increased free-carrier concentration, leading to enhanced carrier scattering and the onset of metallic-like behaviour. Consequently, free-carrier effects partially suppress interband optical transitions, resulting in lower optical absorption than that observed for the 3% oxygen-deficient structure. These results demonstrate that a moderate oxygen-vacancy concentration provides the optimum balance between defect-induced electronic transitions and structural stability, thereby maximizing the optical absorption.These results indicate that a moderate oxygen-vacancy concentration provides the optimum balance between defect-state formation and electronic transport, thereby maximizing optical absorption while preserving the semiconducting characteristics of SnO_2_ [34].

#### 3.2.6. Surface reflectance vs wavelength.

**Fig 7:**
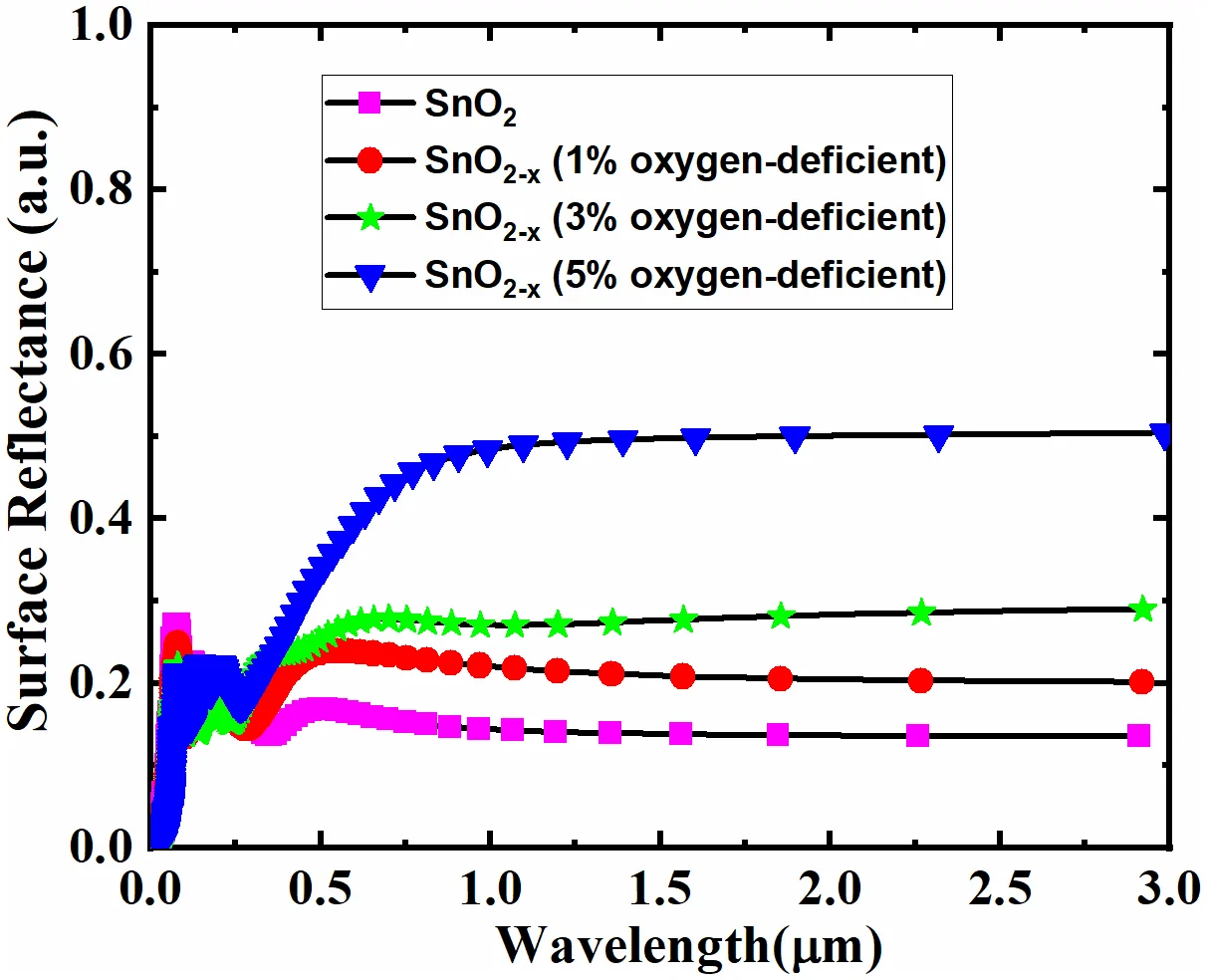
Surface reflectance spectra of SnO_2_ and oxygen deficent SnO_2-x_ configurations.

The wavelength-dependent surface reflectance spectra of pristine SnO_2_ and oxygen-deficient SnO_2-x_ is shown in Fig 7. Surface reflectance represents the fraction of incident electromagnetic radiation reflected from the material surface and is governed by the complex dielectric function, refractive index, and extinction coefficient.

Pristine SnO_2_ exhibits relatively low reflectance throughout the investigated wavelength range, increasing slightly from approximately 0.05 at short wavelengths to about 0.14–0.15 beyond 0.8 μm, which is characteristic of its wide-band-gap semiconducting nature. The introduction of oxygen vacancies modifies the surface reflectance by altering the electronic structure and dielectric response of the material. The 1% oxygen-deficient SnO_2_ shows a moderate increase in reflectance, reaching approximately 0.20–0.22 at wavelengths above 0.8 μm, whereas the 3% oxygen-deficient SnO_2_ exhibits a further increase to approximately 0.27–0.29, indicating enhanced interaction between incident electromagnetic radiation and the vacancy-modified electronic states [37]. This behaviour is consistent with the increase in the refractive index and dielectric constant observed for the oxygen-deficient structures.

A more pronounced enhancement is observed for the 5% oxygen-deficient SnO_2_, where the reflectance increases rapidly with wavelength and approaches a nearly constant value of approximately 0.50 beyond 1.0 μm. This substantial increase is attributed to the high concentration of oxygen vacancies, which introduces a large density of donor-like defect states and free carriers, significantly modifying the low-energy dielectric response. The increased carrier concentration enhances the reflection of incident radiation and is indicative of the onset of metallic-like optical behaviour under heavy oxygen deficiency. However, this increase in reflectance is accompanied by the reduction in the extinction coefficient and absorption coefficient observed for the 5% oxygen-deficient structure, suggesting that excessive oxygen vacancies promote free-carrier reflection and carrier scattering rather than efficient optical absorption. Therefore, although the 5% oxygen-deficient model exhibits the highest reflectance, the 3% oxygen-deficient SnO_2_ provides a more balanced optical response by combining enhanced dielectric behaviour with stronger optical absorption, making it more suitable for optoelectronic and hydrogen gas-sensing applications [38].

### 3.3. H_2_ gas adsorption and sensing behavior

Hydrogen adsorption have been investigated on the thermodynamically stable SnO_2_(110) surface using the Adsorption Locator module of Materials Studio [39]. The Adsorption Locator employs a Monte Carlo-based search algorithm to identify energetically favourable adsorption sites by exploring multiple molecular orientations and surface configurations, thereby predicting the most stable adsorption geometries. Monte Carlo sampling algorithm with the COMPASS II force field, evaluates both van der Waals and electrostatic interactions.

**Fig 8:**
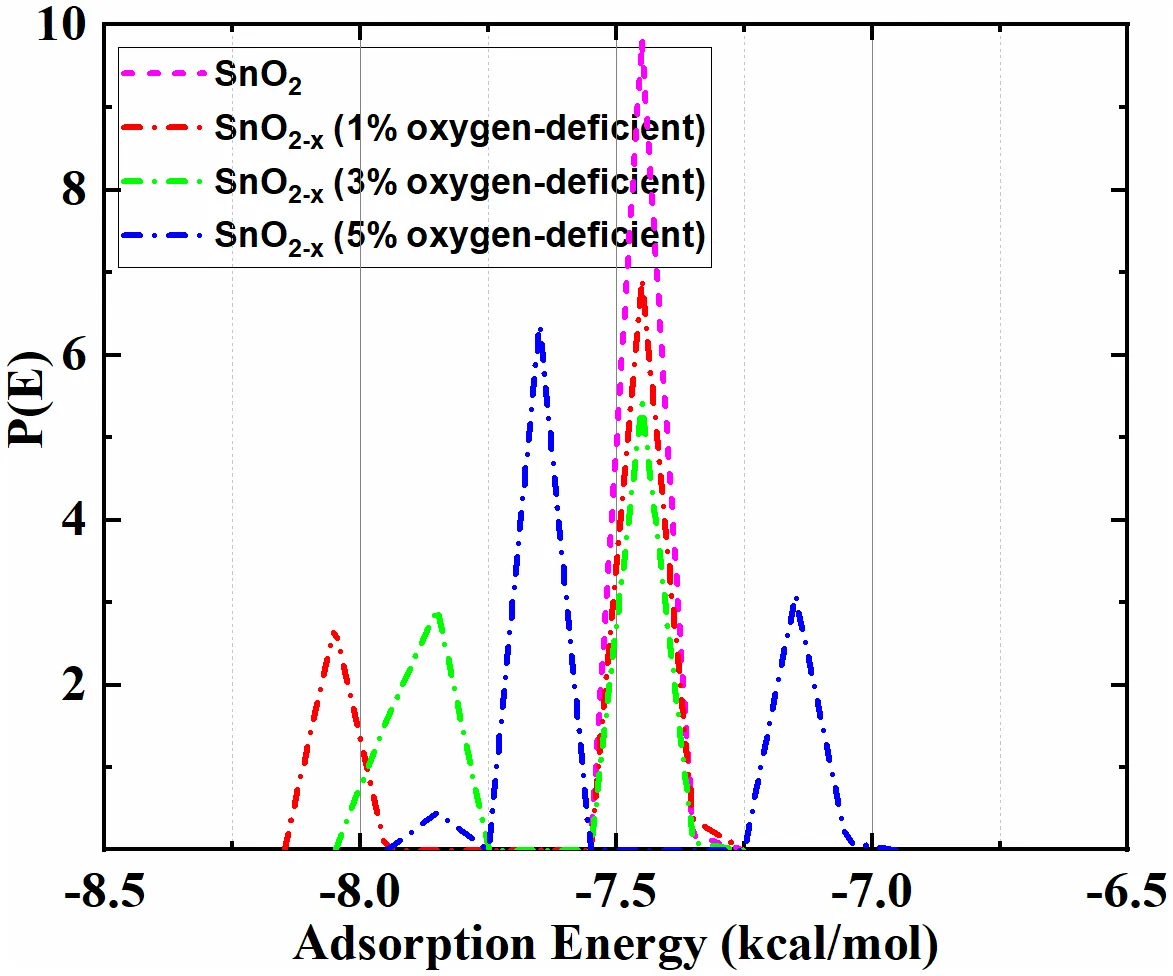
Probability distribution of H_2_ adsorption energies on pristine SnO_2_ and oxygen-deficient SnO_2-x_ surfaces.

The SnO_2_(110) surface was selected because it possesses the lowest surface energy among the low-index rutile SnO_2_ surfaces and is therefore the most stable and experimentally relevant surface for gas-sensing applications [40]. Oxygen vacancies are introduced into the surface layer to evaluate their influence on H_2_ adsorption behaviour. The Fig 8 represents the probability distribution (P(E)) of finding an adsorption site of H_2_ with a specific adsorption energy on pristine and oxygen-deficient SnO_2_(110) surfaces. More negative adsorption energies correspond to stronger interactions between the H_2_ molecule and the surface, while the probability distribution reflects the availability and stability of different adsorption configurations [41].

The pristine SnO_2_(110) surface exhibits a sharp distribution centred at approximately −7.5 kcal mol^−1^ with a P(E) of nearly 9.8, indicating that hydrogen adsorption primarily occurs at a single energetically favourable adsorption site. Upon introducing oxygen vacancies, the adsorption-energy distributions become broader, demonstrating that oxygen vacancies generate additional adsorption sites with different local electronic environments [42]. The 1% oxygen-deficient SnO_2_(110) surface exhibits an additional peak near −8.0 kcal mol^−1^ with a P(E) of ~2.6, suggesting the formation of stronger adsorption sites associated with oxygen-vacancy defects. For the 3% oxygen-deficient surface, the adsorption-energy distribution extends over a wider energy range, with the principal peak remaining near −7.5 kcal mol^−1^ and a secondary peak around −7.8 kcal mol^−1^, indicating that a moderate oxygen-vacancy concentration provides a greater number of energetically favourable adsorption configurations. In contrast, the 5% oxygen-deficient SnO_2_(110) surface exhibits a broader bimodal distribution with peaks near −7.7 and −7.1 kcal mol ^−1^, reflecting increased structural disorder and a wider variation in adsorption strengths caused by excessive oxygen vacancies [43]. Although a higher vacancy concentration introduces more active adsorption sites, excessive defects distort the local atomic and electronic structure, resulting in adsorption sites with different binding energies rather than uniformly stronger adsorption [44]. These results indicate that a moderate oxygen-vacancy concentration provides the optimum balance between the creation of active adsorption sites and surface structural stability, thereby enhancing hydrogen adsorption and supporting the improved gas-sensing performance of oxygen-deficient SnO_2-x_ [45].

## 4. Conclusion

This study systematically investigated the influence of oxygen vacancies on the electronic structure, optical properties, and hydrogen adsorption behaviour of SnO_2_ using first-principles DFT calculations. Oxygen-deficient models, SnO_2-x_ (x = 0.02, 0.06, and 0.10), corresponding to 1%, 3%, and 5% oxygen-vacancy concentrations, revealed that oxygen vacancies introduce donor-like defect states near the conduction band, modifying the electronic structure and optical response. Among the investigated models, the 3% oxygen-deficient SnO_2_ exhibited the most favourable overall performance, with a maximum absorption coefficient of approximately 7 × 10⁴ cm^−1^, an extinction coefficient of about 2.6, and enhanced H_2_ adsorption through the creation of energetically favourable active sites. In contrast, the 5% oxygen-deficient SnO_2_ exhibited a high dielectric constant (Re(ε) > 25), a refractive index approaching 5.4, and a surface reflectance of approximately 0.50, indicating the onset of metallic-like optical behaviour accompanied by increased carrier scattering and structural disorder. Hydrogen adsorption studies performed on the thermodynamically stable SnO_2_(110) surface using the Adsorption Locator module further confirmed that moderate oxygen-vacancy concentrations provide the optimum balance between electronic structure modification, optical performance, and adsorption characteristics. These findings demonstrate that controlled oxygen-vacancy engineering is an effective strategy for optimizing SnO_2_ for hydrogen gas-sensing and optoelectronic applications while providing valuable theoretical guidance for the design of advanced semiconductor materials.

## Supporting information

S1 File Data. (ZIP)
