# Supplementary material for: Density functional theory investigation of oxygen-deficient SnO2-x: Optoelectronic property tuning and hydrogen gas interaction
Source: PLoS One. 2026 Sep 2;21(9):e0357265. doi: 10.1371/journal.pone.0357265 (PMC13537568; doi:10.1371/journal.pone.0357265)
Supplement: Supplementary file 1 [file pone.0357265.s003.docx]

**Response to Reviewers**

**Manuscript Title:** **Density Functional Theory Investigation of Oxygen-Deficient SnO_2-x_: Optoelectronic Property Tuning and Hydrogen Gas Interaction**

**Manuscript ID:**PONE-D-26-21777

Dear Editor and Reviewers,

We sincerely thanks the Academic Editor and the reviewers for their careful evaluation of our manuscript and for the constructive suggestions. We have revised the manuscript thoroughly in response to all comments. Below, we provide a point-by-point response to each reviewer comment. All changes have been incorporated into the revised manuscript and highlighted accordingly.

**Response to Reviewer #1**

**Comment 1:Reviewer #1:**
*Please explicitly state the specific supercell dimensions used for the model to ensure scientific reproducibility. Defining the exact number of Sn and O atoms for each vacancy concentration would clarify how the 1%, 3%, and 5% ratios were achieved.*

**Response:**
We sincerely thank the reviewer for this valuable suggestion. We agree that explicitly specifying the supercell dimensions and the exact atomic composition of each defective model is essential for scientific reproducibility.

In the revised manuscript, we have now clarified the model construction in detail. The pristine SnO₂ structure was modeled using a **5 × 5 × 1 supercell**, containing **50 Sn atoms and 100 O atoms**. Oxygen-deficient structures were generated by removing **1, 3, and 5 oxygen atoms** from the pristine supercell, yielding defective compositions of **Sn₅₀O₉₉**, **Sn₅₀O₉₇**, and **Sn₅₀O₉₅**, respectively. The oxygen-vacancy concentration was calculated as **Vacancy concentration (%) = (number of removed O atoms / total number of O atoms in the pristine supercell) × 100**. Since the pristine 5 × 5 × 1 supercell contains **100 oxygen atoms**, the removal of **1, 3, and 5 O atoms** corresponds directly to oxygen-vacancy concentrations of **1%, 3%, and 5%**, respectively.

These details have now been explicitly incorporated into the **Computational Methods** section at **Page 3** of the revised manuscript to improve clarity and reproducibility.

**Comment 2:Reviewer #1:**
*There is a discrepancy in Section 3.1 where the text refers to labels (b) through (e) for the DOS analysis, while Figure 2 uses (a) through (d). Please synchronize the textual references with the graphical labels to ensure clarity.*

**Response:**
We thank the reviewer for identifying this inconsistency. In the revised version, the DOS discussion in Section 3.1 is now fully consistent with the subfigure labels presented in Figure 2 at **Page 7**.

**Comment 3:Reviewer #1:**
*The manuscript reports a dramatic surge in refractive index and extinction coefficient at 3 μm for the 3% oxygen-deficient system. The authors should provide a deeper physical explanation of how increased carrier density from vacancies drives this “plasmon-like” behavior in the infrared region.*

**Response:**
We thank the reviewer for this important comment. In the revised manuscript, we have included the discussion of the anomalous infrared optical response of the oxygen-deficient SnO₂ system, especially for the 3% oxygen-vacancy configuration.

Our interpretation is based on the fact that oxygen vacancies in SnO₂ act as donor-type defects and can increase the electron concentration by introducing vacancy-related states close to the conduction band. Several studies on defective SnO₂ and transparent conducting oxides have shown that oxygen deficiency can increase the n-type carrier density and modify the low-energy dielectric response in the infrared regime. In particular, at sufficiently high carrier concentrations, the free-carrier contribution to the dielectric function becomes important, leading to a strong increase in the imaginary part of the dielectric response at low photon energies and a corresponding change in the refractive index and extinction coefficient.

Accordingly, we now explain the enhanced optical response near **3 μm** in the 3% oxygen-deficient SnO₂ model as follows: increasing oxygen-vacancy concentration introduces donor/defect states and raises the density of defect-associated carriers, which strengthens **low-energy intraband or defect-assisted electronic excitations** in the infrared region. This additional carrier contribution alters the complex dielectric function, and hence can produce a pronounced increase in the calculated refractive index and extinction coefficient in the mid-infrared. We therefore interpret the feature as a **vacancy-induced enhancement of low-energy optical response arising from defect-mediated carrier contributions and free-carrier dielectric screening**, rather than claiming a true localized plasmon resonance.

These discussions have now been included at **Page 12 and Page 13** of the revised manuscript.

**Comment 4:Reviewer #1:**
*The study notes that 5% oxygen deficiency leads to a slight degradation in performance due to increased disorder and scattering. It is recommended to clarify whether this concentration induces significant structural lattice distortion or if the degradation is primarily limited to electronic transport.*

**Response:**

We thank the reviewer for this valuable suggestion. In the revised manuscript, we have clarified the discussion of the **5% oxygen-deficient SnO₂ model** by explicitly distinguishing between the **structural consequences of higher vacancy concentration** and the **electronic/transport-related degradation** induced by these defects.

Specifically, it is observed that increasing the oxygen-vacancy concentration to 5% is expected to affect the system through **two coupled mechanisms**. First, from a structural point of view, the removal of additional oxygen atoms produces a stronger **local perturbation of the Sn–O coordination environment**, which can lead to relaxation of neighboring Sn atoms, modification of bond lengths/angles, and a larger deviation from the ideal rutile-like lattice around the vacancy sites. In oxygen-deficient SnO₂, such vacancies are known to generate local lattice relaxation rather than a complete collapse of the crystal framework, unless the defect concentration becomes sufficiently high to destabilize. Therefore, in the present 5% vacancy model, the effect is more appropriately described as **enhanced local lattice distortion and structural disorder around the defect region**, rather than a definitive long-range structural phase transformation. This interpretation is consistent with prior theoretical studies showing that oxygen vacancies in SnO₂ introduce localized structural relaxation and defect-induced perturbation of the surrounding lattice.

It is more importantly for the observed degradation in calculated performance, a higher vacancy concentration also introduces stronger **electronic disorder**. As the vacancy density increases, defect-related states become more numerous and may broaden or overlap, thereby perturbing the band-edge electronic structure, enhancing carrier localization/scattering, and reducing the effectiveness of charge transport pathways. In this sense, the deterioration at 5% oxygen deficiency is not attributed solely to geometric distortion, but also to the fact that excessive vacancy concentration can push the system from a beneficial “defect-assisted tuning” regime into a more disordered regime where **defect scattering, trap-assisted recombination, and reduced carrier mobility** become increasingly important. This behavior is consistent with the broader understanding of oxygen-deficient SnO₂, where moderate oxygen vacancy concentrations can improve conductivity or optical response, while excessive defect densities tend to introduce disorder and degrade transport-related properties.

These discussions have now been included at **Page 10 and Page 12** of the revised manuscript.

**Responses to Reviewer #2**

**Comment 1:Reviewer #2:**
*The authors state: “SnO₂₋ₓ (x = 0.01, 0.03, 0.05) which corresponds to 1%, 3%, and 5% oxygen vacancy concentrations.” This statement is incorrect. Since each SnO₂ formula unit contains 2 oxygen atoms, x = 0.01 actually corresponds to an oxygen vacancy concentration of 0.01/2 = 0.5%. Please verify and correct the relevant statements throughout the text.*

**Response:**
We sincerely thank the reviewer for pointing out this important issue. We agree that the notation used in the original manuscript was ambiguous and could lead to confusion between the **stoichiometric parameter (x)** in SnO₂₋ₓ and the **oxygen-vacancy concentration (%)**.

In the revised manuscript, we have now clarified the model construction in detail. The pristine SnO₂ structure was modeled using a 5 × 5 × 1 supercell, containing 50 Sn atoms and 100 O atoms. Oxygen-deficient structures were generated by removing 1, 3, and 5 oxygen atoms from the pristine supercell, yielding defective compositions of Sn₅₀O₉₉, Sn₅₀O₉₇, and Sn₅₀O₉₅, respectively. The oxygen-vacancy concentration was calculated as Vacancy concentration (%) = (number of removed O atoms / total number of O atoms in the pristine supercell) × 100. Since the pristine 5 × 5 × 1 supercell contains 100 oxygen atoms, the removal of 1, 3, and 5 O atoms corresponds directly to oxygen-vacancy concentrations of 1%, 3%, and 5%, respectively.

Therefore, in the SnO_2-x_ notation, the defective supercells correspond to x = 0.02, x =0.06, and x =0.10, rather than (x = 0.01), 0.03, and 0.05. Since each SnO_2_ formula unit contains two oxygen atoms, the oxygen-vacancy concentration relative to the oxygen sublattice is (x/2), i.e., (0.02/2 = 0.01), (0.06/2 = 0.03), and (0.10/2 = 0.05), corresponding to 1%, 3%, and 5% oxygen-vacancy concentrations, respectively.

We have corrected this notation throughout the revised manuscript and now explicitly distinguish between oxygen-vacancy concentration and the stoichiometric parameter (x) to avoid ambiguity.

These discussions have now been included at **Page 3** of the revised manuscript.

**Comment 2:Reviewer #2:**

*The text mentions “supercell containing approximately 100 oxygen atoms.” The word “approximately” is unacceptable for a DFT calculation paper. Please specify the exact dimensions of the supercell (e.g., a 3×3×2 supercell containing 36 Sn atoms + 72 O atoms) and the total number of atoms.*

**Response:**
We thank the reviewer for this important comment and fully agree that the use of the word “approximately” is inappropriate in the context of a DFT study, where the structural model must be defined exactly and unambiguously.

In the revised manuscript, we have removed this imprecise wording and now explicitly specify the exact supercell dimensions, atomic composition, and total number of atoms used in the calculations. The pristine SnO₂ model was constructed using a 5 × 5 × 1 supercell, which contains 50 Sn atoms and 100 O atom. In the revised manuscript, we have now clarified the model construction in detail. The pristine SnO₂ structure was modeled using a 5 × 5 × 1 supercell, containing 50 Sn atoms and 100 O atoms. Oxygen-deficient structures were generated by removing 1, 3, and 5 oxygen atoms from the pristine supercell, yielding defective compositions of Sn₅₀O₉₉, Sn₅₀O₉₇, and Sn₅₀O₉₅, respectively. The oxygen-vacancy concentration was calculated as Vacancy concentration (%) = (number of removed O atoms / total number of O atoms in the pristine supercell) × 100. Since the pristine 5 × 5 × 1 supercell contains 100 oxygen atoms, the removal of 1, 3, and 5 O atoms corresponds directly to oxygen-vacancy concentrations of 1%, 3%, and 5%, respectively. When normalized per Sn atom, these correspond to the compositions SnO₁.₉₈, SnO₁.₉₄, and SnO₁.₉₀, respectively.

These discussions have now been included at **Page 3** of the revised manuscript.

**Comment 3:Reviewer #2:**
*Multiple oxygen vacancies can adopt various spatial configurations, and the calculation results are sensitive to the configuration. The text does not discuss the following key issues: (a) the basis for selecting the vacancy arrangement; (b) whether configurational averaging was performed; (c) whether the vacancies are distributed in a clustered or dispersed manner. Please supplement the relevant explanations.*

**Response:**
We thank the reviewer for this important and constructive comment. We agree that, in oxygen-deficient oxide systems, the calculated structural, electronic, and optical properties can depend on the **specific spatial arrangement of the oxygen vacancies**, especially when more than one vacancy is introduced into a finite periodic supercell. We have therefore revised the manuscript to clarify the basis for selecting the vacancy configurations used in the present study, the spatial distribution of the vacancies, and the scope and limitations of the configurational sampling.

In the revised manuscript, the oxygen vacancy positions were chosen as representative oxygen-deficient configurations while avoiding unrealistically strong vacancy–vacancy interactions caused by placing vacancies immediately adjacent to one another within the periodic supercell. This choice was motivated by the fact that oxygen vacancies in SnO₂ can exhibit **site dependence, defect–defect interaction, and concentration-dependent stability**, and different vacancy geometries may lead to different local lattice relaxations and electronic states. In the present calculations, the vacancy sites for the multi-vacancy models were chosen to be **relatively dispersed rather than strongly clustered**, so as to reduce unrealistically strong local vacancy–vacancy coupling within the finite periodic cell and to better represent a dilute-to-moderate oxygen-deficient regime. At the same time, we acknowledge that at higher oxygen deficiency, local vacancy association or short-range ordering can become energetically relevant in reducible oxides, and alternative arrangements, including more clustered configurations may lead to somewhat different defect formation energies, local structural distortions, and electronic/optical responses.

We further acknowledge that the present study **does not perform configurational averaging** over all symmetry-inequivalent vacancy distributions for the 3% and 5% oxygen-deficient models. Such an analysis would require systematic enumeration of vacancy arrangements, total-energy ranking of multiple defective supercells, and ideally thermodynamic/configurational averaging methods such as cluster-expansion or Monte Carlo approaches, which are beyond the scope of the present work. Instead, the goal of this study was to compare the trends in structural, electronic, and optical properties of pristine SnO₂ with a small set of **representative oxygen-deficient models** at increasing vacancy concentration. We have now made this limitation explicit in the revised manuscript and have moderated the discussion accordingly, emphasizing that the reported results should be interpreted as the behavior of **selected representative vacancy configurations** rather than a complete configurational average over all possible oxygen-vacancy arrangements.

These discussions have now been included at **Page 3** of the revised manuscript.

**Comment 4:Reviewer #2:**
*The charge state of the oxygen vacancies is not specified. Are the oxygen vacancies treated as neutral, +1, or +2? Oxygen vacancies (especially the neutral VO⁰) may produce unpaired electrons, which requires spin-polarized calculations. No related discussion appears in the text; please supplement this.*

**Response:**
We thank the reviewer for this important observation. We agree that the charge state of oxygen vacancies and the possible role of spin polarization should be stated explicitly, particularly because oxygen vacancies in SnO₂ can introduce defect-related electronic states that may influence the calculated electronic and optical properties.

In the present study, the oxygen-deficient SnO₂ systems are modeled using **neutral supercells**, and therefore the oxygen vacancies were treated as neutral oxygen vacancies within the periodic DFT framework rather than as explicitly charged defects such as V_O_^+^ or V_O_^2+^. This choice is consistent with the scope of the present work, which is focused on comparing the structural, electronic, and optical behavior of representative oxygen-deficient SnO₂ models at different vacancy concentrations, rather than performing a full charged-defect thermodynamic analysis.

We also agree with the reviewer that neutral oxygen vacancies may, in principle, introduce **defect-localized electrons** and potentially give rise to **spin-polarized states**. To account for this possibility, **spin-polarized calculations were performed for all vacancy-containing systems**, and this has now been explicitly clarified in the revised **Computational Methods** section. The revised manuscript also now states clearly that the reported defect states, band-structure modifications, and optical-property changes in the oxygen-deficient SnO₂ models correspond to **neutral oxygen-vacancy configurations treated within a spin-polarized DFT framework**. We believe this clarification makes the defect treatment and the computational assumptions used in the present study more transparent and scientifically rigorous.

These discussions have now been included at **Page 4** of the revised manuscript.

**Comment 5:Reviewer #2:**

*Calculating the oxygen vacancy formation energy Ef(VO) is one of the basic requirements for DFT defect studies, but it is completely absent from this paper. In addition, no criterion for structural stability is provided.*

**Response:**
We sincerely thank the reviewer for this valuable comment. We agree that the oxygen-vacancy formation energy, E_f_(V_O_), is an important parameter in DFT defect studies because it provides information on the energetic feasibility of defect formation and the thermodynamic stability of defective structures.

In the revised manuscript, to evaluate the energetic feasibility of oxygen-vacancy formation and confirm the stability of the constructed SnO₂₋ₓ models, the oxygen-vacancy formation energies have benn calculated for the different defect concentrations. The formation energies which are found out to be 3.1 eV, 2.7 eV, and 2.3 eV for the 1%, 3%, and 5% oxygen vacancy models, respectively. These values are in good agreement with previously reported DFT studies, which typically report oxygen-vacancy formation energies. The positive formation energies indicate that all oxygen-deficient configurations are energetically feasible. The slight decrease in formation energy with increasing vacancy concentration suggests that the formation of additional vacancies becomes progressively more favorable because of defect-defect interactions and local structural relaxation. Furthermore, all structures were fully optimized until the convergence criteria for total energy, forces, and stresses were satisfied. The optimized structures retained the rutile SnO₂ framework without any structural collapse, although local distortions around the vacancy sites became more pronounced at higher vacancy concentrations. These results demonstrate that the investigated oxygen-deficient SnO₂ models are both energetically and structurally stable, supporting the reliability of the calculated electronic and optical properties.

These discussions have now been included at **Page 5** of the revised manuscript.

**Comment 6:Reviewer #2:**

*The authors use the GGA-PBE functional and obtain a band gap of “~3.6 eV” for pristine SnO₂. This result is inconsistent with existing literature and theoretical common knowledge: a large body of published work shows that the PBE functional yields a SnO₂ band gap of only about 1.0–1.3 eV. Please verify the calculation setup and provide a reasonable explanation for this significant discrepancy.*

**Response:**
We sincerely thank the reviewer for pointing out this important issue. We agree that the original statement was incorrect and resulted from a typographical error in the manuscript. The value of **~3.6 eV** quoted for pristine SnO₂ does **not** correspond to our GGA-PBE calculation; rather, it is the **experimental band gap** reported in the literature for rutile SnO₂. In the revised manuscript, this statement has been corrected accordingly.

These discussions have now been included at **Page 8** of the revised manuscript.

**Comment 7:Reviewer #2:**
*The DOS evidence is insufficient. The total density of states (DOS) plot alone cannot prove that the defect states are contributed by interstitial oxygen. It is recommended to provide the projected density of states (PDOS) to support the related conclusions.*

**Response:**
We thank the reviewer for this constructive suggestion. In response, we have revised the discussion accordingly and, in the revised manuscript, we have **added projected density of states (PDOS) analysis.**

Additional Figure 3, showing the PDOS plots, has now been included at Page 10 of the revised manuscript.

**Comment 8:Reviewer #2**
*The logical chain between Section 3.3 and the preceding sections is broken. The DOS calculations in Section 3.1 appear to be based on the bulk structure, whereas the H₂ adsorption in Section 3.3 necessarily involves a surface system. There is no clear logical connection between the two systems; please clarify.*

**Response:**
We sincerely thank the reviewer for this insightful comment. We appreciate this helpful observation and agree that the distinction between the bulk and surface calculations, as well as their logical connection, should be explained more clearly.

In the revised manuscript, we have rewritten the relevant parts of both the **Computational Methods** and **Results and Discussion** sections to explicitly distinguish between the two types of calculations performed in this study: (i) bulk supercell calculations, which were carried out to investigate the influence of oxygen vacancies on the structural, electronic, and optical properties of SnO₂, and (ii) surface slab calculations, which were employed to evaluate the H₂ adsorption behavior relevant to gas-sensing applications.

We now clarify that the bulk calculations provide fundamental insight into how oxygen vacancies modify the intrinsic electronic structure of SnO₂, including the introduction of defect-induced donor states near the Fermi level, enhanced carrier concentration, and changes in the optical response. These electronic modifications constitute the underlying physical basis for the improved surface activity of oxygen-deficient SnO₂. Although the adsorption calculations were performed using a surface model, the surface electronic properties are intrinsically linked to the bulk defect chemistry because oxygen vacancies generated in the bulk or near-surface region alter the local electronic environment and promote charge transfer at surface adsorption sites. Consequently, the bulk density of states (DOS) analysis establishes the electronic origin of vacancy-induced changes, whereas the surface adsorption calculations demonstrate how these changes influence the interaction between H₂ molecules and the SnO₂ surface.

This approach is consistent with previous first-principles studies, which have shown that bulk oxygen vacancies introduce donor-like defect states that enhance the chemical reactivity and adsorption characteristics of oxide semiconductor surfaces. Therefore, the bulk electronic structure analysis and the surface adsorption study are complementary rather than independent investigations, providing a comprehensive understanding of the role of oxygen vacancies in determining both the intrinsic material properties and the gas-sensing performance of SnO₂.

These discussions have now been included at **Page 5** of the revised manuscript.

**Comment 9:Reviewer #2**
*Key parameters for H₂ surface adsorption are missing. H₂ adsorption is essentially a surface behavior, but the text does not provide the following key information: (a) the crystal plane used (the (110), (101), and (100) surfaces of SnO₂ have different properties); (b) the thickness of the slab model, the number of atomic layers, and the vacuum layer thickness; (c) whether the surface is Sn-terminated or O-terminated. Please supplement each of these.*

**Response:**
We sincerely thank the reviewer for this valuable comment. We agree that the structural details of the surface model are essential for the reproducibility and interpretation of the H₂ adsorption calculations. In the revised manuscript, we have expanded the **Computational Methods** section to explicitly describe the surface model employed in this study.

Specifically, the H₂ adsorption calculations were carried out on the **SnO₂ (110)** surface, which is the most thermodynamically stable and widely investigated surface of rutile SnO₂ for gas-sensing applications. The surface was modeled using a slab consisting of **five atomic layers**, separated by a vacuum region of 15 Å to eliminate interactions between periodically repeated slabs. The exposed surface was, and the adsorption calculations were performed after full structural relaxation of the slab and the adsorbed H₂ molecule.

These details have now been explicitly included in the revised manuscript. The choice of the (110) surface is consistent with numerous experimental and first-principles studies demonstrating that it is the lowest-energy and most stable surface of rutile SnO₂ and therefore the most relevant model for investigating gas adsorption and sensing mechanisms.

These discussions have now been included at **Page 7 and Page 20** of the revised manuscript.

**Comment 10:Reviewer #2**
*“Fig. 2 (a–d) illustrate the total density of states for (b) pristine SnO₂, (c)…” Please verify and correct the subfigure lettering.*

**Response:**
We thank the reviewer for identifying this inconsistency. The subfigure lettering of *Fig. 2 (a–d)* have been carefully corrected in the revised manuscript at **Page 9.**

**Comment 11:Reviewer #2**

*The version of the Grimme dispersion correction is not specified. Is the dispersion correction D2, D3, D3(BJ), or D4? Different versions have a significant impact on the results; please clarify.*

**Response:**
We sincerely thank the reviewer for this valuable comment. We agree that the specific version of the Grimme dispersion correction should be explicitly stated to ensure the transparency and reproducibility of the computational methodology. In the revised **Computational Methods** section, we have now specified that the calculations were performed using the **Grimme D3(BJ)** scheme.

The inclusion of the D3(BJ) correction is particularly important for the H₂ adsorption calculations because the interaction between H₂ molecules and the SnO₂ surface involves not only covalent and electrostatic contributions but also weak long-range van der Waals (dispersion) interactions. Conventional GGA-PBE functionals are known to underestimate these dispersion forces, which can affect the predicted adsorption energies, equilibrium adsorption distances, and adsorption configurations. By incorporating the Grimme D3(BJ) correction, these long-range interactions are more accurately described, leading to a more reliable representation of the H₂–SnO₂ adsorption system. The corresponding information has now been explicitly included in the revised manuscript to provide a complete, transparent, and reproducible description of the computational methodology.

These discussions have now been included at **Page 5** of the revised manuscript.

**Comment 12:Reviewer #2:**
*The calculated refractive index n ≈ 15 is clearly too high and exceeds the reasonable range for conventional semiconductor materials. Please carefully check the calculation procedure and parameter settings to confirm the reliability of the results.*

**Response:**

We sincerely thank the reviewer for this careful observation. . In response to the reviewer's comment, we carefully re-examined the optical-property calculations, verified the computational parameters, and repeated the post-processing procedure.

Following this verification, the refractive-index calculations were corrected, and the revised results are now consistent with the expected optical behavior of SnO₂ and with values reported in the literature. The corresponding figure has been replaced with the corrected data, and the discussion has been revised accordingly to reflect the updated results.

These discussions have now been included at **Page 13 and Page 14** of the revised manuscript.

**Comment 13:Reviewer #2:**
*For a supercell of about 100 atoms, a 2×2×2 Γ-centered k-point mesh may be acceptable for geometry optimization, but it is insufficient for optical-constant calculations (which require a much higher k-point density than SCF) and for DOS smoothness. A k-point convergence test is recommended, and the results should be reported in the text.*

**Response:**
We sincerely thank the reviewer for this valuable suggestion. We agree that denser k-point sampling is desirable for obtaining highly converged density of states (DOS) and optical properties. The calculations in the present work were performed using a Γ-centered 2 × 2 × 2 Monkhorst–Pack k-point mesh, which was selected as a compromise between computational cost and accuracy for the relatively large 5 × 5 × 1 SnO₂ supercell. This k-point density is commonly employed for geometry optimization and electronic structure calculations of large oxide supercells, where Brillouin-zone folding substantially reduces the k-point sampling required relative to primitive cells. primitive cells. We acknowledge that a denser k-point mesh could further improve the smoothness of the DOS and the convergence of the optical constants.

This limitation has now been explicitly stated in the revised manuscript, and a note has been added indicating that a more extensive k-point convergence study will be considered in future work.

These discussions have now been included at **Page 5** of the revised manuscript.

**Comment 14:Reviewer #2:**
*The conclusion section is severely redundant. It is recommended to consolidate and condense the relevant content.*

**Response:**
We appreciate this suggestion. The **Conclusion** section has been carefully revised .

These discussions have now been included at **Page 22** of the revised manuscript.

**Comment 15:Reviewer #2:**
*Fig. 1 lacks lattice parameters and crystallographic direction labels, and the vacancy markers (white circles) have poor visibility. In Fig. 2, the Fermi level is not indicated on the DOS plots, and in several panels the curve labels overlap with the legend. Please improve these.*

**Response:**
We sincerely thank the reviewer for these valuable suggestions regarding the presentation of the figures. In the revised manuscript, all relevant figures have been carefully revised to improve their clarity, readability, and overall presentation.

The Figure 1and Figure 2 has been reformatted with improved labels, and annotation, at **Page 4 and Page 9,** respectively.

**Comment 16:Reviewer #2:**
*Fig. 5 shows an extremely strong absorption peak in the absorption-coefficient plot in the wavelength range 0.0–0.1 µm. However, this range is already close to the extreme ultraviolet region, and the physical plausibility is questionable. Please check whether this is a numerical artifact.*

**Response:**
We sincerely thank the reviewer for this careful observation. In response, we thoroughly re-examined the optical-property calculations, including the dielectric-function data, post-processing procedure, and the generation of the absorption spectra.

Our investigation confirmed that the unusually strong absorption peak observed in the wavelength range of ~0.0 μm was not a physically meaningful feature but originated from numerical noise introduced during the calculation and post-processing near the zero-wavelength (high-energy) limit. The corresponding figure has been revised and replaced with the corrected absorption spectrum, which now exhibits a physically consistent optical response. The discussion in the manuscript has also been updated to reflect the corrected results. These revisions have now been incorporated into the revised manuscript.

These discussions have now been included at **Page 17** of the revised manuscript.

**Responses to Reviewer #3**

**Comment 1:Reviewer #3:**
*The definition of x in SnO₂₋ₓ and its relationship with the stated 1%, 3%, and 5% oxygen vacancy concentrations should be clarified. The authors should specify the actual numbers of Sn and O atoms in the supercell, the number of removed O atoms, and the actual stoichiometry of each defective model.*

**Response:**
We sincerely thank the reviewer for this valuable suggestion. We agree that explicitly specifying the supercell dimensions and the exact atomic composition of each defective model is essential for scientific reproducibility.

In the revised manuscript, we have now clarified the model construction in detail. The pristine SnO₂ structure was modeled using a 5 × 5 × 1 supercell, containing 50 Sn atoms and 100 O atoms. Oxygen-deficient structures were generated by removing 1, 3, and 5 oxygen atoms from the pristine supercell, yielding defective compositions of Sn₅₀O₉₉, Sn₅₀O₉₇, and Sn₅₀O₉₅, respectively. The oxygen-vacancy concentration was calculated as Vacancy concentration (%) = (number of removed O atoms / total number of O atoms in the pristine supercell) × 100. Since the pristine 5 × 5 × 1 supercell contains 100 oxygen atoms, the removal of 1, 3, and 5 O atoms corresponds directly to oxygen-vacancy concentrations of 1%, 3%, and 5%, respectively. Therefore, in the SnO_2-x_ notation, the defective supercells correspond to x = 0.02, x =0.06, and x =0.10. Since each SnO_2_ formula unit contains two oxygen atoms, the oxygen-vacancy concentration relative to the oxygen sublattice is (x/2), i.e., (0.02/2 = 0.01), (0.06/2 = 0.03), and (0.10/2 = 0.05), corresponding to 1%, 3%, and 5% oxygen-vacancy concentrations, respectively.

We have corrected this notation throughout the revised manuscript and now explicitly distinguish between oxygen-vacancy concentration and the stoichiometric parameter (x) to avoid ambiguity.

These discussions have now been included at **Page 3** of the revised manuscript.

**Comment 2:Reviewer #3**
*More details are needed on the construction of oxygen vacancy models. The authors should clarify whether the vacancies were introduced in bulk or surface models, how the vacancy sites were selected, whether different vacancy configurations were compared, and whether the vacancies marked in Figure 1 represent the most stable configurations.*

**Response:**
We sincerely thank the reviewer for this valuable comment. In the revised manuscript, we have included the description of the oxygen-vacancy model construction to improve the clarity and reproducibility of the computational methodology. We now explicitly state that the oxygen vacancies used for the structural, electronic, and optical property calculations were introduced into the **bulk 5 × 5 × 1 SnO₂ supercell** containing 50 Sn and 100 O atoms. Oxygen-deficient structures were generated by removing **1, 3, and 5 oxygen atoms** from the pristine supercell, yielding defective compositions of **Sn₅₀O₉₉**, **Sn₅₀O₉₇**, and **Sn₅₀O₉₅**, respectively. The oxygen-vacancy concentration was calculated as **Vacancy concentration (%) = (number of removed O atoms / total number of O atoms in the pristine supercell) × 100**. Since the pristine 5 × 5 × 1 supercell contains **100 oxygen atoms**, the removal of **1, 3, and 5 O atoms** corresponds directly to oxygen-vacancy concentrations of **1%, 3%, and 5%**, respectively.Therefore, in the SnO_2-x_ notation, the defective supercells correspond to x = 0.02, x =0.06, and x =0.10. Since each SnO_2_ formula unit contains two oxygen atoms, the oxygen-vacancy concentration relative to the oxygen sublattice is (x/2), i.e., (0.02/2 = 0.01), (0.06/2 = 0.03), and (0.10/2 = 0.05), corresponding to 1%, 3%, and 5% oxygen-vacancy concentrations, respectively.

In the present calculations, the vacancy sites for the multi-vacancy models were chosen to be relatively dispersed rather than strongly clustered, so as to reduce unrealistically strong local vacancy–vacancy coupling within the finite periodic cell and to better represent a dilute-to-moderate oxygen-deficient regime. Furthermore, the oxygen-deficient SnO₂ systems are modeled using **neutral supercells**, and therefore the oxygen vacancies were treated as neutral oxygen vacancies within the periodic DFT framework rather than as explicitly charged defects such as V_O_^+^ or V_O_^2+^. This choice is consistent with the scope of the present work, which is focused on comparing the structural, electronic, and optical behavior of representative oxygen-deficient SnO₂ models.

These discussions have now been included at **Page 3** and **Page 4** of the revised manuscript.

**Comment 3:Reviewer #3:**
*The manuscript mainly presents TDOS results. It is suggested that PDOS analysis be added to clarify whether the defect states mainly originate from Sn 5s/5p or O 2p orbitals, which would strengthen the discussion of vacancy-induced electronic and optical changes.*

**Response:**
We thank the reviewer for this valuable suggestion. In response, we have added **PDOS Plot at Fig 3** and PDOS analysis has been now included in the revised manuscript at **Page 10, 11 and 12.**

**Comment 4:Reviewer #3:**
*The H₂ adsorption section requires more methodological details, including the surface facet, vacuum thickness, surface size, H₂ coverage, sampling method, force field or energy evaluation method used in Adsorption Locator, and the adsorption energy formula. The authors should also clarify whether DFT-optimized adsorption energies were calculated. Since the adsorption energy difference between pristine SnO₂ and defective SnO₂ is relatively small, statements such as “significantly enhanced adsorption strength” should be softened.*

**Response:**

We sincerely thank the reviewer for this valuable comment. We agree that additional methodological details are necessary to improve the transparency and reproducibility of the H₂ adsorption calculations. In the revised manuscript, we have expanded the **Computational Methods** section to clearly describe the adsorption model, including the surface facet, slab dimensions, vacuum thickness, surface supercell size, H₂ coverage, and the computational settings adopted in the **Adsorption Locator** module. We have also specified the sampling algorithm, the force field used for evaluating adsorption configurations, and the adsorption energy expression employed in this study. Furthermore, we now explicitly clarify that the adsorption energies reported in the manuscript correspond to the optimized adsorption configurations obtained using the adopted computational protocol.

In response to the reviewer's observation, we have revised the discussion of the adsorption results. Hydrogen adsorption have been investigated on the thermodynamically stable SnO₂(110) surface using the Adsorption Locator module of Materials Studio. The Adsorption Locator employs a Monte Carlo-based search algorithm to identify energetically favourable adsorption sites by exploring multiple molecular orientations and surface configurations, thereby predicting the most stable adsorption geometries. Monte Carlo sampling algorithm with the COMPASS II force field, evaluates both van der Waals and electrostatic interactions.

The SnO₂(110) surface was selected because it possesses the lowest surface energy among the low-index rutile SnO₂ surfaces and is therefore the most stable and experimentally relevant surface for gas-sensing applications. Oxygen vacancies are introduced into the surface layer to evaluate their influence on H₂ adsorption behaviour. The Fig 8 represents the probability distribution of H₂ adsorption energies on pristine and oxygen-deficient SnO₂(110) surfaces. More negative adsorption energies correspond to stronger interactions between the H₂ molecule and the surface, while the probability distribution reflects the availability and stability of different adsorption configurations.

The pristine SnO₂(110) surface exhibits a sharp distribution centred at approximately −7.5 kcal mol⁻¹ with a maximum probability of nearly 9.8, indicating that hydrogen adsorption primarily occurs at a single energetically favourable adsorption site. Upon introducing oxygen vacancies, the adsorption-energy distributions become broader, demonstrating that oxygen vacancies generate additional adsorption sites with different local electronic environments. The 1% oxygen-deficient SnO₂(110) surface exhibits an additional peak near −8.0 kcal mol⁻¹ with a probability of approximately 2.6, suggesting the formation of stronger adsorption sites associated with oxygen-vacancy defects. For the 3% oxygen-deficient surface, the adsorption-energy distribution extends over a wider energy range, with the principal peak remaining near −7.5 kcal mol⁻¹ and a secondary peak around −7.8 kcal mol⁻¹, indicating that a moderate oxygen-vacancy concentration provides a greater number of energetically favourable adsorption configurations. In contrast, the 5% oxygen-deficient SnO₂(110) surface exhibits a broader bimodal distribution with peaks near −7.7 and −7.1 kcal mol⁻¹, reflecting increased structural disorder and a wider variation in adsorption strengths caused by excessive oxygen vacancies. Although a higher vacancy concentration introduces more active adsorption sites, excessive defects distort the local atomic and electronic structure, resulting in adsorption sites with different binding energies rather than uniformly stronger adsorption. These results indicate that a moderate oxygen-vacancy concentration provides the optimum balance between the creation of active adsorption sites and surface structural stability, thereby enhancing hydrogen adsorption and supporting the improved gas-sensing performance of oxygen-deficient SnO₂ .

These discussions have now been included at **Page 20, 21 and 22** of the revised manuscript.

**Comment 5:Reviewer #3::**
*The manuscript should be carefully checked for formatting and language issues. For example, “SNO2-x” should be uniformly written as “SnO₂₋ₓ”, and the description of Figure 2 should be corrected because the text and panel labels are inconsistent.*

**Response:**
We thank the reviewer for this comment. The revised manuscript has been carefully proofread to correct formatting, notation, and language issues throughout the text. In particular, the notation of **SnO₂₋ₓ** has been standardized consistently, and the Figure 2 description has been corrected to match the actual panel labels.

**Comment 6:Reviewer #3:**
*It is suggested that Figure 1 be revised using a white background for better clarity.*

**Response:**
We thank the reviewer for this helpful suggestion. **Figure 1** has been revised to improve visual clarity, including the background and the visibility of the oxygen-vacancy markers, so that the structural models can be interpreted more easily.
