# Supplementary material for: Density functional theory investigation of oxygen-deficient SnO2-x: Optoelectronic property tuning and hydrogen gas interaction
Source: PLoS One. 2026 Sep 2;21(9):e0357265. doi: 10.1371/journal.pone.0357265 (PMC13537568; doi:10.1371/journal.pone.0357265)
Supplement: Supplementary file 2 [file pone.0357265.s004.docx]

**Response to Reviewers**

**Manuscript Title:** **Density Functional Theory Investigation of Oxygen-Deficient SnO_2-x_: Optoelectronic Property Tuning and Hydrogen Gas Interaction**

**Manuscript ID: PONE-D-26-21777R1**

Dear Editor and Reviewers,

We sincerely thanks the Academic Editor and the reviewers for their careful evaluation of our manuscript and for the constructive suggestions. We have revised the manuscript thoroughly in response to all comments. Below, we provide a point-by-point response to each reviewer comment. All changes have been incorporated into the revised manuscript and highlighted accordingly.

**Response to Reviewer #2**

**Comment 1:Reviewer #2:**
*The authors used a 2×2×2 K-point mesh for the 5×5×1 supercell model. However, this setting is physically unreasonable. Since the supercell is not expanded along the c axis, a uniform 2×2×2 mesh would lead to severe undersampling of the reciprocal space along the c axis. The authors are requested to provide a reasonable justification for this choice and supplement it with convergence tests.*

**Response:**
We sincerely thank the reviewer for pointing out this issue. We apologize for the **typographical error** in reporting the k-point mesh. The k-point mesh was incorrectly mentioned as 2×2×2 in the manuscript. The actual calculations were performed using a **2×2×1 Monkhorst–Pac**k k-point mesh for the 5×5×1 thin-film supercell.

For the thin-film model, the 2×2×1 k-point mesh is appropriate because the supercell is expanded in the in-plane directions, while the c-axis is the out-of-plane direction containing the vacuum region. Thus, no additional k-point sampling along the c-axis was used.

To verify the adequacy of the selected k-point mesh, k-point convergence tests were performed by comparing the calculated total energy and relevant electronic properties using progressively denser meshes. The results showed that the 2 × 2 × 1 mesh provides converged results within the required accuracy, with no significant change in the calculated quantities upon further increasing the k-point density.

The **typographical error** has been corrected at **Page 5** of the revised manuscript.

**Comment 2:Reviewer #2:**
*The manuscript text discusses orbital descriptions of specific elements, such as "Sn 5s and O 2s states". However, the legends in Figure 3 (a–d) only label the orbitals simply as s, p, d, and f, without distinguishing which element (Sn or O) they belong to. If Figure 3 presents the projected density of states (PDOS), it is recommended that the legends or subfigure labels clearly distinguish between the elements and their respective orbitals..*

**Response:**

We sincerely thank the reviewer for pointing out this inconsistency. We have revised the Fig 3(a–d) in the revised manuscript to clearly distinguish the elemental orbital contributions. The generic labels s, p, d, and f have been replaced with the appropriate orbital assignments, namely O-2s + Sn-5s, O-2p + Sn-5p, and Sn-4d.

The manuscript text has also been revised at **Page 9 and Page 10** to maintain consistency with these combined PDOS contributions.

In the revised version, the revised Figure 3 has been included at **Page 10**.
