# Supplementary material for: Density functional theory investigation of oxygen-deficient SnO2-x: Optoelectronic property tuning and hydrogen gas interaction
Source: PLoS One. 2026 Sep 2;21(9):e0357265. doi: 10.1371/journal.pone.0357265 (PMC13537568; doi:10.1371/journal.pone.0357265)
Supplement: Peer review history

# Peer review: Decision Letter 0

**DOI:** 10.1371/journal.pone.0357265.r001

**Authors:** Kelong Fan

9 Jun 2026

Dear Dr. Singh,

Thank you for submitting your manuscript to PLOS ONE. After careful consideration, we feel that it has merit but does not fully meet PLOS ONE’s publication criteria as it currently stands. Therefore, we invite you to submit a revised version of the manuscript that addresses the points raised during the review process.

Please submit your revised manuscript by Aug 08 2026 11:59PM. If you will need more time than this to complete your revisions, please reply to this message or contact the journal office at plosone@plos.org. When you're ready to submit your revision, log on to https://www.editorialmanager.com/pone/ and select the 'Submissions Needing Revision' folder to locate your manuscript file.

- A letter that responds to each point raised by the academic editor and reviewer(s). You should upload this letter as a separate file labeled 'Response to Reviewers'.
- A marked-up copy of your manuscript that highlights changes made to the original version. You should upload this as a separate file labeled 'Revised Manuscript with Track Changes'.
- An unmarked version of your revised paper without tracked changes. You should upload this as a separate file labeled 'Manuscript'.

If you would like to make changes to your financial disclosure, please include your updated statement in your cover letter. Guidelines for resubmitting your figure files are available below the reviewer comments at the end of this letter.

If applicable, we recommend that you deposit your laboratory protocols in protocols.io to enhance the reproducibility of your results. Protocols.io assigns your protocol its own identifier (DOI) so that it can be cited independently in the future. For instructions see: https://journals.plos.org/plosone/s/submission-guidelines#loc-laboratory-protocols. Additionally, PLOS ONE offers an option for publishing peer-reviewed Lab Protocol articles, which describe protocols hosted on protocols.io. Read more information on sharing protocols at https://plos.org/protocols?utm_medium=editorial-email&utm_source=authorletters&utm_campaign=protocols.

As the corresponding author, your ORCID iD is verified in the submission system and will appear in the published article. PLOS supports the use of ORCID, and we encourage all coauthors to register for an ORCID iD and use it as well. Please encourage your coauthors to verify their ORCID iD within the submission system before final acceptance, as unverified ORCID iDs will not appear in the published article. *Only*  the individual author can complete the verification step; PLOS staff *cannot*  verify ORCID iDs on behalf of authors.

We look forward to receiving your revised manuscript.

Kind regards,

Kelong Fan

Academic Editor

PLOS One

Journal Requirements:

When submitting your revision, we need you to address these additional requirements.

1. Please ensure that your manuscript meets PLOS ONE's style requirements, including those for file naming. The PLOS ONE style templates can be found at

https://journals.plos.org/plosone/s/file?id=wjVg/PLOSOne_formatting_sample_main_body.pdf and

https://journals.plos.org/plosone/s/file?id=ba62/PLOSOne_formatting_sample_title_authors_affiliations.pdf

2. Please note that PLOS One has specific guidelines on code sharing for submissions in which author-generated code underpins the findings in the manuscript. In these cases, we expect all author-generated code to be made available without restrictions upon publication of the work. Please review our guidelines at https://journals.plos.org/plosone/s/materials-and-software-sharing#loc-sharing-code and ensure that your code is shared in a way that follows best practice and facilitates reproducibility and reuse.

3. When completing the data availability statement of the submission form, you indicated that you will make your data available on acceptance. We strongly recommend all authors decide on a data sharing plan before acceptance, as the process can be lengthy and hold up publication timelines. Please note that, though access restrictions are acceptable now, your entire data will need to be made freely accessible if your manuscript is accepted for publication. This policy applies to all data except where public deposition would breach compliance with the protocol approved by your research ethics board. If you are unable to adhere to our open data policy, please kindly revise your statement to explain your reasoning and we will seek the editor's input on an exemption. Please be assured that, once you have provided your new statement, the assessment of your exemption will not hold up the peer review process.

4. PLOS requires an ORCID iD for the corresponding author in Editorial Manager on papers submitted after December 6th, 2016. Please ensure that you have an ORCID iD and that it is validated in Editorial Manager. To do this, go to ‘Update my Information’ (in the upper left-hand corner of the main menu), and click on the Fetch/Validate link next to the ORCID field. This will take you to the ORCID site and allow you to create a new iD or authenticate a pre-existing iD in Editorial Manager.

5. If the reviewer comments include a recommendation to cite specific previously published works, please review and evaluate these publications to determine whether they are relevant and should be cited. There is no requirement to cite these works unless the editor has indicated otherwise.

[Note: HTML markup is below. Please do not edit.]

Reviewers' comments:

Reviewer's Responses to Questions

**Comments to the Author**

1. Is the manuscript technically sound, and do the data support the conclusions?

Reviewer #1: Yes

Reviewer #2: No

Reviewer #3: Yes

**********

2. Has the statistical analysis been performed appropriately and rigorously?

Reviewer #1: Yes

Reviewer #2: No

Reviewer #3: Yes

**********

3. Have the authors made all data underlying the findings in their manuscript fully available?

The PLOS Data policy

Reviewer #1: Yes

Reviewer #2: No

Reviewer #3: Yes

**********

4. Is the manuscript presented in an intelligible fashion and written in standard English?

Reviewer #1: Yes

Reviewer #2: Yes

Reviewer #3: Yes

**********

Reviewer #1: The manuscript presents a systematic Density Functional Theory (DFT) study on the impact of oxygen vacancies (Ov) on the electronic, optical, and adsorption properties of tetragonal rutile SnO2. The authors successfully modeled four systems: pristine SnO2 and oxygen-deficient SnO2-x at concentrations of 1%, 3%, and 5%. The study identifies a critical threshold at 3% vacancy concentration, where the material exhibits optimized optoelectronic responses. The findings regarding H2 gas interaction provide useful theoretical insights into the role of defect engineering for sensor enhancement. The methodology is technically sound, utilizing the CASTEP module with a high plane-wave cutoff energy of 830 eV and PBE exchange-correlation functionals.

1.Please explicitly state the specific supercell dimensions used for the model to ensure scientific reproducibility. Defining the exact number of Sn and O atoms for each vacancy concentration would clarify how the 1%, 3%, and 5% ratios were achieved.

2.There is a discrepancy in Section 3.1 where the text refers to labels (b) through (e) for the DOS analysis, while Figure 2 uses (a) through (d). Please synchronize the textual references with the graphical labels to ensure clarity.

3.The manuscript reports a dramatic surge in refractive index and extinction coefficient at 3μm for the 3% oxygen-deficient system. The authors should provide a deeper physical explanation of how increased carrier density from vacancies drives this “plasmon-like” behavior in the infrared region.

4.The study notes that 5% oxygen deficiency leads to a slight degradation in performance due to increased disorder and scattering. It is recommended to clarify whether this concentration induces significant structural lattice distortion or if the degradation is primarily limited to electronic transport.

Reviewer #2: This manuscript employs first-principles density functional theory (DFT) calculations to systematically investigate the effects of different oxygen vacancy concentrations (1%, 3%, and 5%) on the electronic structure, optical properties, and H₂ gas-sensing performance of SnO₂. The chosen research topic has some scientific value. However, the manuscript exhibits several significant deficiencies in methodological rigor, the reasonableness of key physical quantities, and the reproducibility of the results. Based on an overall evaluation, I recommend rejection. Specific comments are as follows:

1. The authors state: “SnO₂₋ₓ (x = 0.01, 0.03, 0.05) which corresponds to 1%, 3%, and 5% oxygen vacancy concentrations.” This statement is incorrect. Since each SnO₂ formula unit contains 2 oxygen atoms, x = 0.01 actually corresponds to an oxygen vacancy concentration of 0.01/2 = 0.5%. Please verify and correct the relevant statements throughout the text.

2. The text mentions “supercell containing approximately 100 oxygen atoms.” The word “approximately” is unacceptable for a DFT calculation paper. Please specify the exact dimensions of the supercell (e.g., a 3×3×2 supercell containing 36 Sn atoms + 72 O atoms) and the total number of atoms.

3. Multiple oxygen vacancies can adopt various spatial configurations, and the calculation results are sensitive to the configuration. The text does not discuss the following key issues: (a) the basis for selecting the vacancy arrangement; (b) whether configurational averaging was performed; (c) whether the vacancies are distributed in a clustered or dispersed manner. Please supplement the relevant explanations.

4. The charge state of the oxygen vacancies is not specified. Are the oxygen vacancies treated as neutral, +1, or +2? Oxygen vacancies (especially the neutral VO⁰) may produce unpaired electrons, which requires spin-polarized calculations. No related discussion appears in the text; please supplement this.

5. Calculating the oxygen vacancy formation energy Ef(VO) is one of the basic requirements for DFT defect studies, but it is completely absent from this paper. In addition, no criterion for structural stability is provided.

6. The authors use the GGA-PBE functional and obtain a band gap of “~3.6 eV” for pristine SnO₂. This result is inconsistent with existing literature and theoretical common knowledge: a large body of published work shows that the PBE functional yields a SnO₂ band gap of only about 1.0–1.3 eV. Please verify the calculation setup and provide a reasonable explanation for this significant discrepancy.

7. The DOS evidence is insufficient. The total density of states (DOS) plot alone cannot prove that the defect states are contributed by interstitial oxygen. It is recommended to provide the projected density of states (PDOS) to support the related conclusions.

8. The logical chain between Section 3.3 and the preceding sections is broken. The DOS calculations in Section 3.1 appear to be based on the bulk structure, whereas the H₂ adsorption in Section 3.3 necessarily involves a surface system. There is no clear logical connection between the two systems; please clarify.

9. Key parameters for H₂ surface adsorption are missing. H₂ adsorption is essentially a surface behavior, but the text does not provide the following key information: (a) the crystal plane used (the (110), (101), and (100) surfaces of SnO₂ have different properties); (b) the thickness of the slab model, the number of atomic layers, and the vacuum layer thickness; (c) whether the surface is Sn-terminated or O-terminated. Please supplement each of these.

10. “Fig. 2 (a–d) illustrate the total density of states for (b) pristine SnO₂, (c)…” Please verify and correct the subfigure lettering.

11. The version of the Grimme dispersion correction is not specified. Is the dispersion correction D2, D3, D3(BJ), or D4? Different versions have a significant impact on the results; please clarify.

12. The calculated refractive index n ≈ 15 is clearly too high and exceeds the reasonable range for conventional semiconductor materials. Please carefully check the calculation procedure and parameter settings to confirm the reliability of the results.

13. For a supercell of about 100 atoms, a 2×2×2 Γ-centered k-point mesh may be acceptable for geometry optimization, but it is insufficient for optical-constant calculations (which require a much higher k-point density than SCF) and for DOS smoothness. A k-point convergence test is recommended, and the results should be reported in the text.

14. The conclusion section is severely redundant. It is recommended to consolidate and condense the relevant content.

15. Fig. 1 lacks lattice parameters and crystallographic direction labels, and the vacancy markers (white circles) have poor visibility. In Fig. 2, the Fermi level is not indicated on the DOS plots, and in several panels the curve labels overlap with the legend. Please improve these.

16. Fig. 5 shows an extremely strong absorption peak in the absorption-coefficient plot in the wavelength range 0.0–0.1 µm. However, this range is already close to the extreme ultraviolet region, and the physical plausibility is questionable. Please check whether this is a numerical artifact.

Reviewer #3: The manuscript uses density functional theory to investigate the electronic structure, optical properties, and H₂ adsorption behavior of oxygen-deficient SnO₂₋ₓ with 1%, 3%, and 5% oxygen vacancies. The results suggest that moderate oxygen deficiency, especially 3%, may improve the optical response and gas-sensing performance of SnO₂. The topic is relevant, and the computational results provide useful theoretical insights into defect-engineered SnO₂. I recommend publication after minor revision.

1. The definition of x in SnO₂₋ₓ and its relationship with the stated 1%, 3%, and 5% oxygen vacancy concentrations should be clarified. The authors should specify the actual numbers of Sn and O atoms in the supercell, the number of removed O atoms, and the actual stoichiometry of each defective model.

2. More details are needed on the construction of oxygen vacancy models. The authors should clarify whether the vacancies were introduced in bulk or surface models, how the vacancy sites were selected, whether different vacancy configurations were compared, and whether the vacancies marked in Figure 1 represent the most stable configurations.

3. The manuscript mainly presents TDOS results. It is suggested that PDOS analysis be added to clarify whether the defect states mainly originate from Sn 5s/5p or O 2p orbitals, which would strengthen the discussion of vacancy-induced electronic and optical changes.

4. The H₂ adsorption section requires more methodological details, including the surface facet, vacuum thickness, surface size, H₂ coverage, sampling method, force field or energy evaluation method used in Adsorption Locator, and the adsorption energy formula. The authors should also clarify whether DFT-optimized adsorption energies were calculated. Since the adsorption energy difference between pristine SnO₂ and defective SnO₂ is relatively small, statements such as “significantly enhanced adsorption strength” should be softened.

5. The manuscript should be carefully checked for formatting and language issues. For example, “SNO2-x” should be uniformly written as “SnO₂₋ₓ”, and the description of Figure 2 should be corrected because the text and panel labels are inconsistent.

6. It is suggested that Figure 1 be revised using a white background for better clarity.

**********

what does this mean?). If published, this will include your full peer review and any attached files.

If you choose “no”, your identity will remain anonymous but your review may still be made public.

**Do you want your identity to be public for this peer review?**  For information about this choice, including consent withdrawal, please see our Privacy Policy

Reviewer #1: No

Reviewer #2: No

Reviewer #3: No

**********

[NOTE: If reviewer comments were submitted as an attachment file, they will be attached to this email and accessible via the submission site. Please log into your account, locate the manuscript record, and check for the action link "View Attachments". If this link does not appear, there are no attachment files.]

To ensure your figures meet our technical requirements, please review our figure guidelines: https://journals.plos.org/plosone/s/figures

You may also use PLOS’s free figure tool, NAAS, to help you prepare publication quality figures: https://journals.plos.org/plosone/s/figures#loc-tools-for-figure-preparation.

NAAS will assess whether your figures meet our technical requirements by comparing each figure against our figure specifications.

# Author comment: Author response to Decision Letter 1

**DOI:** 10.1371/journal.pone.0357265.r002

14 Jul 2026

Manuscript Title: Density Functional Theory Investigation of Oxygen-Deficient SnO2-x: Optoelectronic Property Tuning and Hydrogen Gas Interaction

Manuscript ID:PONE-D-26-21777

Dear Editor and Reviewers,

We sincerely thanks the Academic Editor and the reviewers for their careful evaluation of our manuscript and for the constructive suggestions. We have revised the manuscript thoroughly in response to all comments. Below, we provide a point-by-point response to each reviewer comment. All changes have been incorporated into the revised manuscript and highlighted accordingly.

Attachment Submitted filename: response to reviewer_pone.docx

# Peer review: Decision Letter 1

**DOI:** 10.1371/journal.pone.0357265.r003

**Authors:** Kelong Fan

9 Aug 2026

Dear Dr. Singh,

Please submit your revised manuscript by Sep 08 2026 11:59PM. If you will need more time than this to complete your revisions, please reply to this message or contact the journal office at plosone@plos.org. When you're ready to submit your revision, log on to https://www.editorialmanager.com/pone/ and select the 'Submissions Needing Revision' folder to locate your manuscript file.

- A letter that responds to each point raised by the academic editor and reviewer(s). You should upload this letter as a separate file labeled 'Response to Reviewers'.
- A marked-up copy of your manuscript that highlights changes made to the original version. You should upload this as a separate file labeled 'Revised Manuscript with Track Changes'.
- An unmarked version of your revised paper without tracked changes. You should upload this as a separate file labeled 'Manuscript'.

If you would like to make changes to your financial disclosure, please include your updated statement in your cover letter. Guidelines for resubmitting your figure files are available below the reviewer comments at the end of this letter.

If applicable, we recommend that you deposit your laboratory protocols in protocols.io to enhance the reproducibility of your results. Protocols.io assigns your protocol its own identifier (DOI) so that it can be cited independently in the future. For instructions see: https://journals.plos.org/plosone/s/submission-guidelines#loc-laboratory-protocols. Additionally, PLOS ONE offers an option for publishing peer-reviewed Lab Protocol articles, which describe protocols hosted on protocols.io. Read more information on sharing protocols at https://plos.org/protocols?utm_medium=editorial-email&utm_source=authorletters&utm_campaign=protocols.

As the corresponding author, your ORCID iD is verified in the submission system and will appear in the published article. PLOS supports the use of ORCID, and we encourage all coauthors to register for an ORCID iD and use it as well. Please encourage your coauthors to verify their ORCID iD within the submission system before final acceptance, as unverified ORCID iDs will not appear in the published article. *Only*  the individual author can complete the verification step; PLOS staff *cannot*  verify ORCID iDs on behalf of authors.

We look forward to receiving your revised manuscript.

Kind regards,

Kelong Fan

Academic Editor

PLOS One

Journal Requirements:

If the reviewer comments include a recommendation to cite specific previously published works, please review and evaluate these publications to determine whether they are relevant and should be cited. There is no requirement to cite these works unless the editor has indicated otherwise.

Please review your reference list to ensure that it is complete and correct. If you have cited papers that have been retracted, please include the rationale for doing so in the manuscript text, or remove these references and replace them with relevant current references. Any changes to the reference list should be mentioned in the rebuttal letter that accompanies your revised manuscript. If you need to cite a retracted article, indicate the article’s retracted status in the References list and also include a citation and full reference for the retraction notice.

[Note: HTML markup is below. Please do not edit.]

Reviewers' comments:

Reviewer's Responses to Questions

**Comments to the Author**

Reviewer #1: All comments have been addressed

Reviewer #2: (No Response)

Reviewer #3: All comments have been addressed

**********

2. Is the manuscript technically sound, and do the data support the conclusions?

Reviewer #1: Yes

Reviewer #2: Partly

Reviewer #3: Yes

**********

3. Has the statistical analysis been performed appropriately and rigorously?

Reviewer #1: Yes

Reviewer #2: I Don't Know

Reviewer #3: Yes

**********

4. Have the authors made all data underlying the findings in their manuscript fully available?

The PLOS Data policy

Reviewer #1: Yes

Reviewer #2: Yes

Reviewer #3: Yes

**********

5. Is the manuscript presented in an intelligible fashion and written in standard English?

Reviewer #1: Yes

Reviewer #2: Yes

Reviewer #3: Yes

**********

Reviewer #1: (No Response)

Reviewer #2: The manuscript has been improved overall, but there are still two specific points that need to be revised and optimized. It is recommended for acceptance after revision.

1. The authors used a 2×2×2 K-point mesh for the 5×5×1 supercell model. However, this setting is physically unreasonable. Since the supercell is not expanded along the c axis, a uniform 2×2×2 mesh would lead to severe under-sampling of the reciprocal space along the c axis. The authors are requested to provide a reasonable justification for this choice and supplement it with convergence tests.

2. The manuscript text discusses orbital descriptions of specific elements, such as "Sn 5s and O 2s states". However, the legends in Figure 3 (a–d) only label the orbitals simply as s, p, d, and f, without distinguishing which element (Sn or O) they belong to. If Figure 3 presents the projected density of states (PDOS), it is recommended that the legends or subfigure labels clearly distinguish between the elements and their respective orbitals.

Reviewer #3: The manuscript has been revised by the author in accordance with the suggested revisions. Acceptance for publication is recommended.

**********

what does this mean?). If published, this will include your full peer review and any attached files.

If you choose “no”, your identity will remain anonymous but your review may still be made public.

**Do you want your identity to be public for this peer review?**  For information about this choice, including consent withdrawal, please see our Privacy Policy

Reviewer #1: No

Reviewer #2: No

Reviewer #3: No

**********

[NOTE: If reviewer comments were submitted as an attachment file, they will be attached to this email and accessible via the submission site. Please log into your account, locate the manuscript record, and check for the action link "View Attachments". If this link does not appear, there are no attachment files.]

To ensure your figures meet our technical requirements, please review our figure guidelines: https://journals.plos.org/plosone/s/figures

You may also use PLOS’s free figure tool, NAAS, to help you prepare publication quality figures: https://journals.plos.org/plosone/s/figures#loc-tools-for-figure-preparation.

NAAS will assess whether your figures meet our technical requirements by comparing each figure against our figure specifications.

# Author comment: Author response to Decision Letter 2

**DOI:** 10.1371/journal.pone.0357265.r004

10 Aug 2026

We sincerely thanks the Academic Editor and the reviewers for their careful evaluation of our manuscript and for the constructive suggestions. We have revised the manuscript thoroughly in response to all comments. Below, we provide a point-by-point response to each reviewer comment. All changes have been incorporated into the revised manuscript and highlighted accordingly.

Attachment Submitted filename: response to reviewer_minor_revision.docx

# Editor report: Decision Letter 2

**DOI:** 10.1371/journal.pone.0357265.r005

**Authors:** Kelong Fan

16 Aug 2026

Density Functional Theory Investigation of Oxygen-Deficient SnO2-x: Optoelectronic Property Tuning and Hydrogen Gas Interaction

PONE-D-26-21777R2

Dear Dr. Singh,

We’re pleased to inform you that your manuscript has been judged scientifically suitable for publication and will be formally accepted for publication once it meets all outstanding technical requirements.

Within one week, you’ll receive an e-mail detailing the required amendments. When these have been addressed, you’ll receive a formal acceptance letter and your manuscript will be scheduled for publication.

An invoice will be generated when your article is formally accepted. Please note, if your institution has a publishing partnership with PLOS and your article meets the relevant criteria, all or part of your publication costs will be covered. Please make sure your user information is up-to-date by logging into Editorial Manager at Editorial Manager® and clicking the ‘Update My Information' link at the top of the page. For questions related to billing, please contact billing support.

If your institution or institutions have a press office, please notify them about your upcoming paper to help maximize its impact. If they’ll be preparing press materials, please inform our press team as soon as possible -- no later than 48 hours after receiving the formal acceptance. Your manuscript will remain under strict press embargo until 2 pm Eastern Time on the date of publication. For more information, please contact onepress@plos.org.

Kind regards,

Kelong Fan

Academic Editor

PLOS One

Additional Editor Comments (optional):

Reviewers' comments:

# Editor report: Acceptance letter

**DOI:** 10.1371/journal.pone.0357265.r006

**Authors:** Kelong Fan

PONE-D-26-21777R2

PLOS One

Dear Dr. Singh,

I'm pleased to inform you that your manuscript has been deemed suitable for publication in PLOS One. Congratulations! Your manuscript is now being handed over to our production team.

At this stage, our production department will prepare your paper for publication. This includes ensuring the following:

* All references, tables, and figures are properly cited

* All relevant supporting information is included in the manuscript submission,

* There are no issues that prevent the paper from being properly typeset

You will receive further instructions from the production team, including instructions on how to review your proof when it is ready. Please keep in mind that we are working through a large volume of accepted articles, so please give us a few days to review your paper and let you know the next and final steps.

Lastly, if your institution or institutions have a press office, please let them know about your upcoming paper now to help maximize its impact. If they'll be preparing press materials, please inform our press team within the next 48 hours. Your manuscript will remain under strict press embargo until 2 pm Eastern Time on the date of publication. For more information, please contact onepress@plos.org.

You will receive an invoice from PLOS for your publication fee after your manuscript has reached the completed accept phase. If you receive an email requesting payment before acceptance or for any other service, this may be a phishing scheme. Learn how to identify phishing emails and protect your accounts at https://explore.plos.org/phishing.

If we can help with anything else, please email us at customercare@plos.org.

Thank you for submitting your work to PLOS One and supporting open access.

Kind regards,

PLOS One Editorial Office Staff

on behalf of

Dr. Kelong Fan

Academic Editor

PLOS One
